# The association between HbA1c/HDL-C ratio and cardiometabolic multimorbidity among middle-aged and older adults: data from two national prospective cohorts in the United Kingdom and United States

**DOI:** 10.3389/fnut.2026.1849520

**Published:** 2026-06-29

**Authors:** Hongxiang Ji, Ziyi Zhao, Ronghao Li, Wenlu Ma, Mimi Zhang, Zhongyun Zhou

**Affiliations:** 1 The First Clinical Medical College, Shandong University of Traditional Chinese Medicine, Jinan, Shandong, China; 2 Department of Hand and Foot, Microsurgery, The Affiliated Hospital of Qingdao University, Qingdao, Shandong, China; 3 Health Examination Center, The Second Affiliated Hospital of Shandong University of Traditional Chinese Medicine, Jinan, Shandong, China; 4 Department of Endocrinology, Jinan Zhangqiu District Hospital of TCM, Jinan, Shandong, China; 5 Department of Cardiology, The Second Affiliated Hospital of Shandong University of Traditional Chinese Medicine, Jinan, Shandong, China

**Keywords:** cardiometabolic multimorbidity, cumulative exposure, ELSA, HbA1c/HDL-C ratio, HRS, prospective cohort study, trajectory analysis

## Abstract

**Objective:**

This study aims to investigate the association between the hemoglobin A1c (HbA1c)/high-density lipoprotein cholesterol (HDL-C) ratio and incident cardiometabolic multimorbidity (CMM) among adults aged 50 years and older.

**Methods:**

This study analyzed data from wave 2 (2004) to wave 11 (2022) of the English Longitudinal Study of Ageing (ELSA) and wave 8 (2006) to wave 16 (2022) of the Health and Retirement Study (HRS). The independent variables included cumulative HbA1c/HDL-C ratio, its value at first visit, and its trajectory identified by group-based trajectory modeling (GBTM). The association between the HbA1c/HDL-C ratio and the risk of CMM was assessed using Kaplan–Meier curves, multivariable Cox regression analysis, and restricted cubic spline (RCS) models. Its incremental predictive value was also evaluated. Additionally, mediation analyses, subgroup analyses, and sensitivity analyses were further employed.

**Results:**

Among the 5,226 participants included, CMM developed in 303 of 1,918 participants (15.8%) in ELSA and in 283 of 3,308 participants (8.8%) in HRS. After adjusting for confounders, each 1-standard deviation (SD) increase in the cumulative HbA1c/HDL-C ratio and the value at first visit HbA1c/HDL-C ratio was significantly associated with a higher risk of CMM [pooled hazard ratio (HR): 1.33, 95% confidence interval (CI): 1.19–1.47, *p* < 0.001; pooled HR: 1.24, 95% CI: 1.07–1.43, *p* = 0.004]. Individuals in the persistently high HbA1c/HDL-C ratio group had a 100% higher risk of developing CMM than those in the persistently low group (pooled HR: 2.00, 95% CI: 1.45–2.74, *p* < 0.001). Linear associations were identified between the cumulative HbA1c/HDL-C ratio and incident CMM in ELSA and HRS, as well as between the HbA1c/HDL-C ratio and incident CMM at first visit in HRS (all *p* for overall <0.05 and *p* for non-linearity >0.05). The addition of the HbA1c/HDL-C ratio significantly improves the predictive ability of the basic model. The relationship between the trajectory of the HbA1c/HDL-C ratio and incident CMM was partially mediated by BMI in HRS. The results from sensitivity analyses and subgroup analyses aligned with the main findings.

**Conclusion:**

Significant linear associations were identified between increases in the HbA1c/HDL-C ratio and the risk of incident CMM among middle-aged and older adults from the United Kingdom (UK) and the United States (US). Persistent monitoring and maintenance of favorable levels of HbA1c/HDL-C ratio may contribute to the early detection and effective prevention of CMM development.

## Graphical abstract

**Figure:**
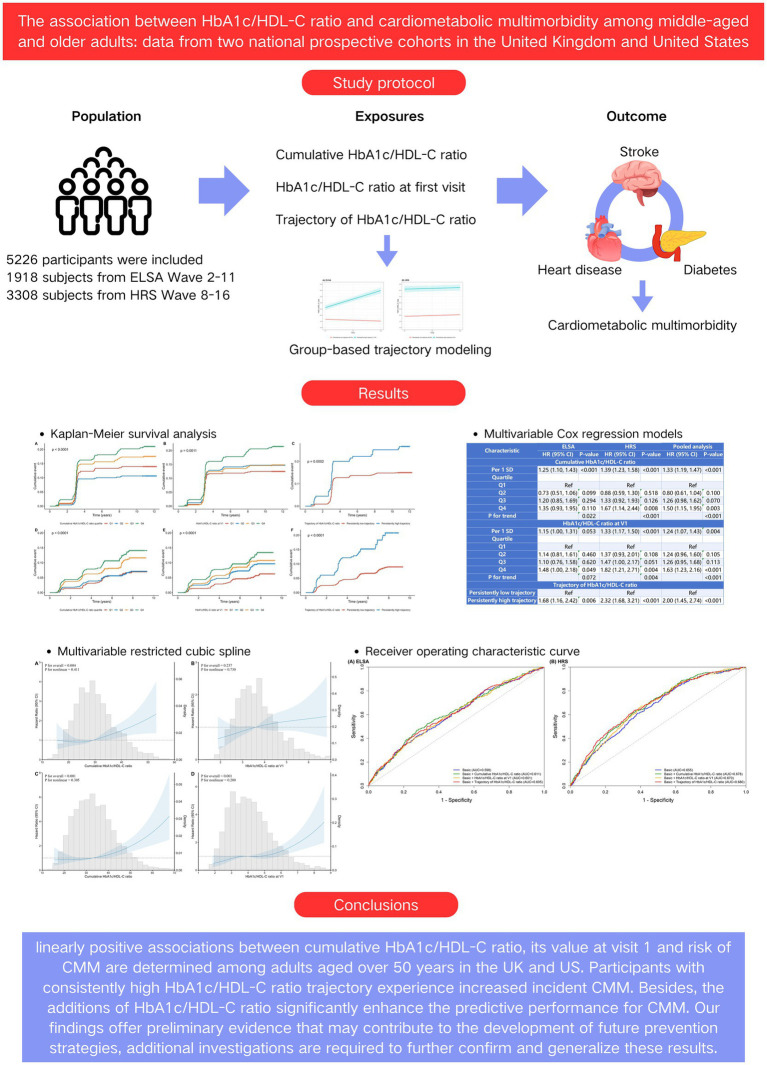

## Introduction

Multimorbidity, defined as the coexistence of two or more chronic conditions within the same individual, represents a major challenge to human health, particularly among middle-aged and older adults (1). Cardiometabolic multimorbidity (CMM), defined as the coexistence of at least two cardiometabolic diseases (CMDs), such as diabetes, heart disease, and stroke, represents one of the most common and serious forms of multimorbidity worldwide (2). Based on data from the National Health and Nutrition Examination Survey (NHANES), Cheng et al. (3) reported that the prevalence of CMM in the US increased significantly between 1999 and 2018, with an average 2-year cycle percentage change of 3.6 (95% CI: 2.1–5.3), reaching 14.4% during 2017–2018 in the US. A growing body of evidence has shown that CMM is closely associated with the onset and progression of a range of chronic conditions, including disability, dementia, cognitive decline, and depression (4–7). In addition, compared with the presence of a single CMD, CMM has been associated with a markedly higher risk of mortality and a substantial reduction in life expectancy (2, 8). Therefore, the early identification and timely management of CMM are essential for promoting healthy aging and alleviating the burden on healthcare systems.

Recent studies have suggested that the ratio of non-high-density lipoprotein cholesterol (non-HDL-C) to HDL-C, as well as the visceral adiposity index, may outperform traditional lipid parameters in predicting CMM (9, 10). However, these markers mainly reflect lipid metabolism, whereas glucose and lipid metabolism are closely interconnected. Evidence indicates that abnormalities in lipid metabolism may not only arise as a consequence of diabetes but may also play a contributory role in the development of glucose metabolism dysfunction (11). The HbA1c/HDL-C ratio was initially introduced by Hu et al. in 2021 (12) as an integrated marker of glucolipid metabolism. Since then, this readily available index has been used to investigate its associations with carotid atherosclerosis (12), diabetic retinopathy (13), metabolic disorders (14), and other related conditions. HbA1c reflects average glycemic levels over the preceding 2 to 3 months and is widely applied in the management of diabetes (15). In addition, previous studies have demonstrated a J-shaped association between HbA1c and both cardiovascular disease (CVD) and all-cause mortality, with HbA1c levels above 8.0% being associated with an elevated risk of CVD and death (16). In contrast, HDL-C exerts multiple biological functions, including reverse cholesterol transport, anti-inflammatory and antithrombotic effects, and the promotion of insulin secretion (17), and has been reported to be inversely associated with the risk of atherosclerotic cardiovascular disease (18).

However, research investigating the relationship between the HbA1c/HDL-C ratio and CMM risk is limited. Therefore, this study uses longitudinal data from two nationwide representative studies to comprehensively assess the associations between the cumulative HbA1c/HDL-C ratio, its value at first visit, and its trajectory and incident CMM among middle-aged and older adults in the UK and the US. Our findings will lay a solid foundation for future in-depth research and clinical applications.

## Methods

### Data sources and study population

Data were derived from the English Longitudinal Study of Aging (ELSA) and the Health and Retirement Study (HRS), two prospective, nationally representative, longitudinal cohorts conducted biennially among adults aged 50 years and older from the UK and the US, respectively (19, 20). Supplementary methods contain more related information. Ethics approval for these surveys was obtained from the relevant institutional review boards, and written informed consent was provided by all participants prior to participation.

Five waves in both cohorts were set as baseline, which were simplified as five visits, i.e., visit 1 to visit 5. In the ELSA, data from wave 2 (2004) to wave 11 (2022) were used, with the time interval between wave 2 (2004) and wave 6 (2012) as the baseline, as the blood biomarkers were examined at wave 2 (2004), wave 4 (2008), and wave 6 (2012). In the HRS, data from wave 8 (2006) to wave 16 (2022) were used, with the time interval between wave 8 (2006)/wave 9 (2008) and wave 12 (2014)/wave 13 (2016) as the baseline, considering that biomarkers data were obtained from one half of the participants in wave 8 (2006), wave 10 (2010), and wave 12 (2014), whereas collection from the remaining half was conducted in wave 9 (2008), wave 11 (2012), and wave 13 (2016). The timepoints of examinations are shown in Supplementary Figure S1.

**Figure 1:**
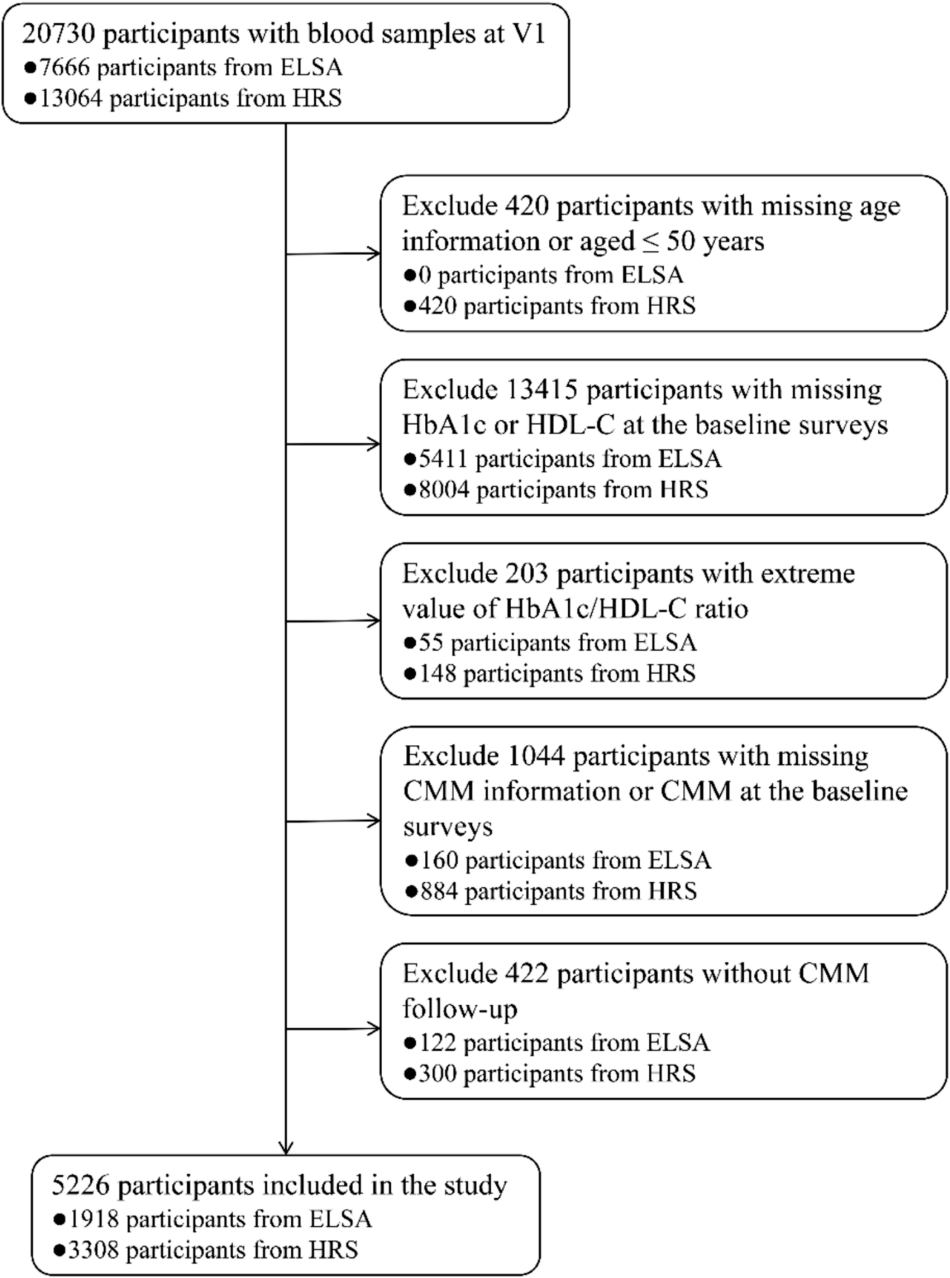
Flowchart of the study population. V1, visit 1; CMM, cardiometabolic multimorbidity.

Initially, 20,730 participants with blood samples were enrolled at visit 1 (7,666 individuals from ELSA and 13,064 from HRS). After excluding 420 participants with missing age data or aged 50 years and younger, 13,415 participants with missing data of HbA1c or HDL-C during visit 1 to visit 5, 203 participants with HbA1c/HDL-C ratio exceeding three standard deviations (SDs) from the mean, 1,044 participants with missing CMM information or CMM between visit 1 and visit 5, and 442 participants without CMM follow-up, 5,226 participants were retained (1,918 from ELSA and 3,308 from HRS) (Figure 1).

### Assessment of HbA1c/HDL-C ratio

The independent variables included cumulative HbA1c/HDL-C ratio, its value at visit 1, and its trajectory. The calculation of HbA1c/HDL-C ratio was based on HbA1c/HDL-C ratio = HbA1c (%)/HDL-C (mmol/L) (21, 22). The cumulative HbA1c/HDL-C ratio was calculated as an area-under-the-curve estimation (mean value * time span) by the formula: cumulative HbA1c/HDL-C ratio = (HbA1c/HDL-C ratio_visit1_ + HbA1c/HDL-C ratio_visit3_)/2*time interval (visit 1 - visit 3) + (HbA1c/HDL-C ratio_visit3_ + HbA1c/HDL-C ratio_visit5_)/2*time interval (visit 3 - visit 5) (23–25). Visit 1, visit 3, and visit 5 indicated three waves, which were wave 2 (2004), wave 4 (2008), and wave 6 (2012) in ELSA, and wave 8 (2006)/wave 9 (2008), wave 10 (2010)/wave 11 (2012), and wave 12 (2014)/wave 13 (2016) in HRS, respectively.

### Determination of CMM

In this study, the outcome was the occurrence of CMM, defined as the simultaneous presence of two or more CMDs, including heart disease, stroke, or diabetes (25, 26). Diagnoses of heart disease and stroke were based on self-reported physician diagnoses obtained through standardized survey questions (“Have you been diagnosed of heart attack, coronary heart disease, angina, congestive heart failure, or other heart problems by a physician?” and “Have you been diagnosed of stroke by a physician?”), supplemented with information on relevant medication use. Diabetes was identified using a combination of biochemical criteria (fasting plasma glucose [FPG] ≥ 126 mg/dL or HbA1c ≥ 6.5%), current use of hypoglycemic drugs, or self-reported physician diagnoses in response to the question “Have you been diagnosed of diabetes or high blood sugar by a physician?” The follow-up period was defined as the duration from baseline to the first recorded occurrence of CMM.

### Collection of covariates

Covariates included age, sex, education, marital status, residence, smoking, drinking, body mass index (BMI), waist circumference (WC), height, weight, systolic blood pressure (SBP), diastolic blood pressure (DBP), dyslipidemia, dyslipidemia medications, hypertension, hypertension medications, diabetes medications, cancer, total cholesterol (TC), low-density lipoprotein cholesterol (LDL-C), triglycerides (TG), fasting plasma glucose (FPG), and C-reactive protein (CRP). Education level was categorized into below high school, high school, and above high school. Marital status was classified as married or other. Residence was categorized as urban and rural. Smoking status and drinking status were each classified as never and ever. BMI was calculated by weight (kg)/height (m)^2^. SBP and DBP were the mean values based on three blood pressure measurements. Dyslipidemia was defined as the presence of any of the following: TG ≥ 150 mg/dL, TC ≥ 240 mg/dL, HDL-C < 40 mg/dL, LDL-C ≥ 160 mg/dL, use of lipid-lowering medications, or a self-reported physician diagnosis of dyslipidemia. Hypertension was considered present if at least one of the following criteria was met: SBP ≥ 140 mmHg, DBP ≥ 90 mmHg, a self-reported physician diagnosis of hypertension, or current use of antihypertensive drugs. Cancer was identified based on a self-reported physician diagnosis. Additionally, residence status and dyslipidemia medications were not applicable in ELSA, whereas LDL-C, TG, and FPG were not tested in HRS.

### Statistical analysis

The details of the missing data of this study are shown in Supplementary Tables S1, S2. Missing data were assumed to be missing at random and were therefore handled using multiple imputations by chained equations. All variables included in our study were incorporated into the imputation model, and the results from the imputed datasets were pooled according to Rubin’s rules.

Group-based trajectory modeling (GBTM) is a statistical method used to identify latent subgroups within longitudinal data by grouping individuals who share similar developmental trends over time. In the present study, GBTM was employed to delineate trajectories of the HbA1c/HDL-C ratio across baseline assessments using measurements obtained at visit 1, visit 3, and visit 5. To determine the optimal model, a range of models with one to five trajectory groups was tested, with trajectory shapes specified using linear, quadratic, or cubic polynomial terms. In accordance with the widely accepted criteria proposed by Nagin, model selection was based on the following considerations: (1) lower values of the Bayesian information criterion and Akaike information criterion; (2) an average posterior probability of group membership greater than 0.7 for each trajectory group; (3) no trajectory group containing fewer than 5% of the total participants; and (4) greater entropy together with visually well-separated fitted trajectories (27). Models with one to five trajectory groups were evaluated. Although the three-group model yielded lower AIC and BIC values, one trajectory subgroup accounted for less than 5% of participants in both ELSA (2.24%) and HRS (4.50%), which did not meet our predefined model selection criterion. In addition, models with four or more groups produced very small classes, reduced classification stability, and lower posterior probabilities. Therefore, considering model parsimony, classification quality, and clinical interpretability, the two-group model was selected as the optimal trajectory solution. The model fit statistics are presented in Supplementary Tables S3, S4.

Categorical variables were presented as numbers (percentages) and were compared using the chi-squared test. Continuous variables with a normal distribution were expressed as mean (SD) and compared using analysis of variance, whereas non-normally distributed continuous variables were summarized as median (interquartile range [IQR]) and compared using the Kruskal–Wallis test.

CMM incidence rates were expressed per 1,000 person-years and were calculated by dividing the number of incident CMM events by the total person-years of follow-up and multiplying the result by 1,000, thereby representing the occurrence of CMM in the population over time.

The HbA1c/HDL-C ratio was evaluated both as a continuous variable and as a categorical variable based on quartiles. Kaplan–Meier curves were generated, and log-rank tests were performed to describe and compare the cumulative incidence of CMM across different groups. Potential multicollinearity between HbA1c/HDL-C ratio and other covariates was assessed by examining the variance inflation factors (VIFs). The VIFs of all covariates included in the multivariable model were less than 5 (Supplementary Tables S5–S10). The associations between HbA1c/HDL-C ratio and incident CMM were assessed using Cox proportional hazards regression models. On the basis of the literature review, three models were constructed. Model 1 included adjustment for age and sex. Model 2 was further adjusted for marital status, educational level, smoking status, drinking status, and WC. Model 3 additionally controlled for dyslipidemia, hypertension, cancer, TC, and CRP. Pooled estimates were derived using random-effects meta-analyses, consistent with previous studies (28–30). Heterogeneity in *β* values across cohorts was examined using Cochran’s Q test and the I^2^ statistic. Fully adjusted restricted cubic spline (RCS) regression models were further applied to investigate the potential non-linear relationship between HbA1c/HDL-C ratio and incident CMM.

Furthermore, the incremental predictive value of incorporating the HbA1c/HDL-C ratio into the basic model was assessed using the receiver operating characteristic (ROC) curves, area under the curve (AUC), net reclassification index (NRI), and integrated discrimination improvement (IDI), and its performance was compared with that of the basic model.

Mediation analysis was performed to examine whether BMI, an indicator of overall adiposity, which is closely associated with both dysglycemia and dyslipidemia, mediated the association between the HbA1c/HDL-C ratio and incident CMM. The distribution of mediation effects was estimated on the basis of 1,000 posterior simulations. The average causal mediation effect (ACME), average direct effect (ADE), and proportion mediated were calculated, and their statistical significance was evaluated using the corresponding *p*-values.

In addition, subgroup and interaction analyses were conducted to determine whether the association between HbA1c/HDL-C ratio and incident CMM differed across strata defined by age (<65 years and ≥65 years), sex, education levels, marital status, smoking status, drinking status, BMI classifications (<25 kg/m^2^ and ≥25 kg/m^2^), dyslipidemia, and hypertension.

To improve the robustness of the findings, five sensitivity analyses were performed. First, the analyses were repeated after excluding participants with missing covariates. Second, logistic regression models were additionally applied to examine the association between the HbA1c/HDL-C ratio and incident CMM. Third, participants receiving treatment for heart disease, stroke, diabetes, hypertension, or dyslipidemia at baseline were excluded. Fourth, participants with a follow-up duration of less than 2 years were excluded to address potential reverse causality. Finally, the E-value was calculated to estimate the minimum strength of association that an unmeasured confounder would need to have with both the HbA1c/HDL-C ratio and CMM risk to fully explain the observed association. The E-value was calculated as follows: E = RR + sqrt{RR × (RR − 1)} (31).

A two-sided *p*-value of < 0.05 was considered statistically significant. All statistical analyses were conducted using R (version 4.2.0).

## Results

### Participant characteristics

**Figure 2:**
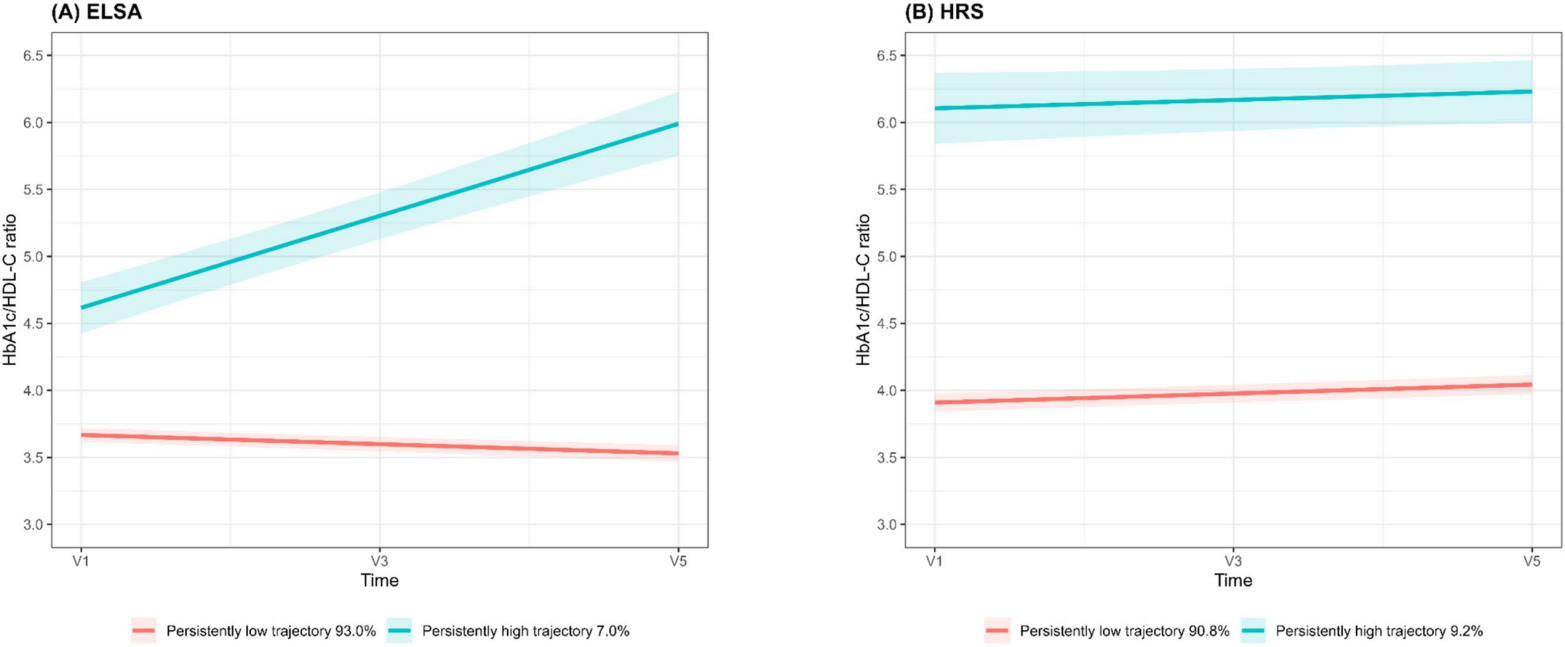
Trajectories of HbA1c/HDL-C ratio during baseline period in ELSA **(A)** and HRS **(B)**. Solid lines are the estimated trajectories, and the shaded bands indicate the corresponding 95% CI. V1, visit 1; V3, visit 3; V5, visit 5.

Ultimately, 5,226 subjects aged 50 years and older were included, with 1,918 from ELSA and 3,308 from HRS. Two optimal distinct HbA1c/HDL-C ratio patterns were identified by GBTM: persistently low (*n* = 1783, 93.0% in ELSA and *n* = 2,907, 90.8% in HRS), which remained at relatively low levels of the HbA1c/HDL-C ratio (<3.75 in ELSA and <4.25 in HRS), and persistently high (*n* = 135, 7.0% in ELSA and *n* = 296, 9.2% in HRS), which maintained a high HbA1c/HDL-C ratio during the baseline period (>4.5 in ELSA and > 6 in HRS) (Figure 2).

The median age of individuals was 61.00 years (IQR: 57.00–68.00 years) in ELSA and 65.00 years (IQR: 58.00–71.00 years) in HRS, with 817 (42.6%) men in ELSA and 1,149 (35.9%) men in HRS. The mean HbA1c/HDL-C ratio was 3.69 (SD: 0.96) in ELSA and 4.16 (SD: 1.30) in HRS at visit 1, 3.86 (SD: 1.03) in ELSA and 4.25 (SD: 1.33) in HRS at visit 3, and 3.68 (SD: 1.16) in ELSA and 4.29 (SD: 1.35) in HRS at visit 5. The mean cumulative HbA1c/HDL-C ratio was 30.16 (SD: 7.77) in ELSA and 33.93 (SD: 9.54) in HRS. During a median follow-up period of 11.58 years in ELSA and 7.08 years in HRS, CMM occurred in 303 (15.8%) participants in ELSA and 283 (8.8%) participants in HRS.

According to quartiles of cumulative HbA1c/HDL-C ratio, participants in the highest quartile were more likely to be men, married, smokers, non-drinkers, prone to dyslipidemia, hypertension, diabetes, had higher BMI, WC, height, weight, HbA1c, CRP, TG, and FPG, and lower TC, LDL-C, and HDL-C in ELSA, compared with other quartiles. In the HRS, participants in the Q4 group tended to be younger, men, married, non-drinkers, had lower education levels, TC, HDL-C, and higher BMI, WC, height, weight, HbA1c, CRP, proportion of dyslipidemia, hypertension, and diabetes (Table 1). Furthermore, baseline characteristics were stratified and summarized according to quartiles of HbA1c/HDL-C ratio at visit 1 and its trajectory (Supplementary Tables S11,S12). Comparisons of baseline characteristics between participants included in and excluded from the analysis in each cohort are presented in Supplementary Table S13.

**Table 1:** Patient demographics and baseline characteristics based on quartiles of cumulative HbA1c/HDL-C ratio.

Characteristics	ELSA						HRS					
	Overall (*N* = 1,918)	Q1: 12.7–24.6 (*N* = 480)	Q2: 24.6–29.3 (*N* = 479)	Q3: 29.3–34.7 (*N* = 479)	Q4: 34.7–57.7 (*N* = 480)	*p*-value	Overall (*N* = 3,203)	Q1: 13.7–26.9 (*N* = 801)	Q2: 26.9–33.0 (*N* = 801)	Q3: 33.0–39.5 (*N* = 801)	Q4: 39.6–74.9 (*N* = 800)	*p*-value
Age	61.00 (57.00, 68.00)	60.50 (56.00, 67.00)	61.00 (57.00, 68.00)	61.00 (57.00, 67.00)	62.00 (57.00, 69.00)	0.319	65.00 (58.00, 71.00)	64.00 (58.00, 70.00)	66.00 (59.00, 72.00)	65.00 (58.00, 71.00)	64.00 (58.00, 71.00)	0.005
Sex						<0.001						<0.001
Male	817 (42.6)	106 (22.1)	148 (30.9)	254 (53.0)	309 (64.4)		1,149 (35.9)	166 (20.7)	246 (30.7)	333 (41.6)	404 (50.5)	
Female	1,101 (57.4)	374 (77.9)	331 (69.1)	225 (47.0)	171 (35.6)		2,054 (64.1)	635 (79.3)	555 (69.3)	468 (58.4)	396 (49.5)	
Education						0.523						<0.001
Below high school	1,084 (56.5)	261 (54.4)	269 (56.2)	265 (55.3)	289 (60.2)		434 (13.5)	70 (8.7)	102 (12.7)	114 (14.2)	148 (18.5)	
High school	160 (8.3)	39 (8.1)	38 (7.9)	41 (8.6)	42 (8.8)		1,126 (35.2)	265 (33.1)	257 (32.1)	295 (36.8)	309 (38.6)	
Above high school	674 (35.1)	180 (37.5)	172 (35.9)	173 (36.1)	149 (31.0)		1,643 (51.3)	466 (58.2)	442 (55.2)	392 (48.9)	343 (42.9)	
Marital status						<0.001						0.028
Married	1,390 (72.5)	327 (68.1)	334 (69.7)	349 (72.9)	380 (79.2)		2,255 (70.4)	563 (70.3)	533 (66.5)	577 (72.0)	582 (72.8)	
Others	528 (27.5)	153 (31.9)	145 (30.3)	130 (27.1)	100 (20.8)		948 (29.6)	238 (29.7)	268 (33.5)	224 (28.0)	218 (27.3)	
Residence						NA						0.861
Urban	NA	NA	NA	NA	NA		2,141 (66.8)	544 (67.9)	528 (65.9)	537 (67.0)	532 (66.5)	
Rural	NA	NA	NA	NA	NA		1,062 (33.2)	257 (32.1)	273 (34.1)	264 (33.0)	268 (33.5)	
Smoking	1,143 (59.6)	274 (57.1)	255 (53.2)	306 (63.9)	308 (64.2)	<0.001	1,665 (52.0)	405 (50.6)	418 (52.2)	419 (52.3)	423 (52.9)	0.825
Drinking	1,775 (92.5)	444 (92.5)	454 (94.8)	448 (93.5)	429 (89.4)	0.010	1,870 (58.4)	556 (69.4)	469 (58.6)	450 (56.2)	395 (49.4)	<0.001
BMI (kg/m^2^)	27.52 (4.53)	25.54 (4.19)	27.31 (4.39)	28.09 (4.16)	29.13 (4.60)	<0.001	28.50 (25.30, 32.30)	26.40 (23.80, 30.10)	28.00 (25.30, 31.80)	29.20 (25.80, 33.50)	30.10 (27.05, 33.90)	<0.001
Waist circumference (cm)	93.81 (12.37)	86.41 (11.31)	91.84 (10.78)	96.35 (11.15)	100.65 (11.52)	<0.001	99.06 (14.67)	91.82 (13.45)	97.42 (13.95)	101.95 (13.75)	105.07 (14.04)	<0.001
Height (m)	1.66 (0.09)	1.63 (0.08)	1.65 (0.09)	1.67 (0.09)	1.69 (0.09)	<0.001	1.65 (0.10)	1.64 (0.10)	1.64 (0.10)	1.66 (0.10)	1.67 (0.10)	<0.001
Weight (kg)	76.21 (14.55)	68.12 (12.12)	74.24 (12.79)	78.87 (13.57)	83.60 (14.89)	<0.001	80.44 (17.38)	73.06 (15.01)	78.22 (16.29)	83.76 (17.57)	86.74 (17.31)	<0.001
SBP (mmHg)	132.57 (17.38)	130.81 (18.56)	133.01 (17.47)	132.86 (16.32)	133.58 (17.02)	0.072	128.65 (19.00)	126.34 (18.77)	128.11 (18.84)	130.32 (20.17)	129.81 (17.94)	<0.001
DBP (mmHg)	76.08 (10.36)	75.17 (10.81)	76.10 (10.04)	76.70 (9.50)	76.35 (11.00)	0.123	79.65 (10.98)	78.72 (10.88)	79.54 (10.80)	80.44 (11.42)	79.90 (10.76)	0.016
Dyslipidemia	1,359 (70.9)	294 (61.3)	324 (67.6)	335 (69.9)	406 (84.6)	<0.001	1,935 (60.4)	431 (53.8)	421 (52.6)	466 (58.2)	617 (77.1)	<0.001
Dyslipidemia treatment	NA	NA	NA	NA	NA	NA	1,128 (35.2)	216 (27.0)	267 (33.3)	314 (39.2)	331 (41.4)	<0.001
Hypertension	927 (48.3)	211 (44.0)	222 (46.3)	233 (48.6)	261 (54.4)	0.007	1,921 (60.0)	409 (51.1)	479 (59.8)	504 (62.9)	529 (66.1)	<0.001
Hypertension treatment	213 (11.1)	44 (9.2)	44 (9.2)	50 (10.4)	75 (15.6)	0.004	1,437 (44.9)	301 (37.6)	347 (43.3)	376 (46.9)	413 (51.6)	<0.001
Diabetes	67 (3.5)	1 (0.2)	4 (0.8)	10 (2.1)	52 (10.8)	<0.001	387 (12.1)	23 (2.9)	53 (6.6)	98 (12.2)	213 (26.6)	<0.001
Diabetes treatment	36 (1.9)	0 (0.0)	1 (0.2)	3 (0.6)	32 (6.7)	<0.001	262 (8.2)	10 (1.2)	35 (4.4)	67 (8.4)	150 (18.8)	<0.001
Heart disease	158 (8.2)	35 (7.3)	37 (7.7)	42 (8.8)	44 (9.2)	0.674	357 (11.1)	89 (11.1)	85 (10.6)	92 (11.5)	91 (11.4)	0.947
Stroke	20 (1.0)	1 (0.2)	5 (1.0)	10 (2.1)	4 (0.8)	0.022	36 (1.1)	6 (0.7)	6 (0.7)	13 (1.6)	11 (1.4)	0.236
Cancer	91 (4.7)	29 (6.0)	28 (5.8)	17 (3.5)	17 (3.5)	0.104	327 (10.2)	84 (10.5)	81 (10.1)	75 (9.4)	87 (10.9)	0.773
TC (mg/dL)	234.03 (44.73)	245.55 (41.83)	240.83 (42.60)	231.92 (46.14)	217.81 (43.31)	<0.001	209.38 (40.05)	224.11 (39.22)	211.59 (37.94)	207.79 (38.49)	194.01 (38.75)	<0.001
HDL-C (mg/dL)	60.48 (14.59)	77.60 (12.22)	63.12 (7.76)	54.99 (7.16)	46.21 (7.56)	<0.001	56.92 (16.16)	74.79 (13.36)	59.62 (10.39)	50.70 (9.67)	42.54 (9.64)	<0.001
HbA1c (%)	5.44 (0.43)	5.29 (0.33)	5.38 (0.34)	5.43 (0.35)	5.65 (0.58)	<0.001	5.65 (0.69)	5.38 (0.42)	5.54 (0.49)	5.65 (0.55)	6.04 (0.99)	<0.001
CRP (ug/mL)	1.70 (0.80, 3.50)	1.10 (0.60, 2.50)	1.70 (0.90, 3.40)	1.90 (0.90, 3.80)	2.30 (1.10, 4.40)	<0.001	1.89 (0.91, 4.27)	1.47 (0.72, 3.27)	1.80 (0.87, 3.74)	2.06 (0.94, 4.98)	2.49 (1.20, 5.47)	<0.001
LDL-C (mg/dL)	143.08 (37.72)	145.62 (36.15)	149.32 (36.59)	145.90 (37.91)	131.49 (37.79)	<0.001	NA	NA	NA	NA	NA	NA
TG (mg/dL)	132.86 (97.43, 186.00)	97.43 (70.86, 132.86)	124.00 (97.43, 177.14)	141.71 (97.43, 186.00)	177.14 (124.00, 239.14)	<0.001	NA	NA	NA	NA	NA	NA
FPG (mg/dL)	89.55 (12.23)	86.75 (10.01)	88.91 (9.89)	89.26 (11.33)	93.29 (15.83)	<0.001	NA	NA	NA	NA	NA	NA
Cumulative HbA1c/HDL-C ratio	30.16 (7.77)	21.17 (2.56)	26.96 (1.38)	31.74 (1.60)	40.78 (4.88)	<0.001	33.93 (9.54)	22.86 (2.85)	30.03 (1.76)	36.08 (1.89)	46.77 (6.47)	<0.001
HbA1c/HDL-C ratio at V1	3.69 (0.96)	2.69 (0.42)	3.34 (0.41)	3.87 (0.47)	4.84 (0.81)	<0.001	4.16 (1.30)	2.87 (0.53)	3.68 (0.61)	4.42 (0.73)	5.67 (1.15)	<0.001
HbA1c/HDL-C ratio at V3	3.86 (1.03)	2.69 (0.37)	3.45 (0.28)	4.08 (0.32)	5.22 (0.74)	<0.001	4.25 (1.33)	2.83 (0.43)	3.73 (0.44)	4.51 (0.52)	5.95 (1.07)	<0.001
HbA1c/HDL-C ratio at V5	3.68 (1.16)	2.50 (0.50)	3.24 (0.44)	3.84 (0.54)	5.12 (0.99)	<0.001	4.29 (1.35)	2.91 (0.58)	3.87 (0.73)	4.59 (0.83)	5.80 (1.14)	<0.001
CMM during follow-up	303 (15.8)	67 (14.0)	51 (10.6)	84 (17.5)	101 (21.0)	<0.001	283 (8.8)	50 (6.2)	52 (6.5)	82 (10.2)	99 (12.4)	<0.001

BMI, body mass index; SBP, systolic blood pressure; DBP, diastolic blood pressure; TC, total cholesterol; HDL-C, high-density lipoprotein cholesterol; CRP, C-reactive protein; LDL-C, low-density lipoprotein cholesterol; TG, triglycerides; FPG, fasting plasma glucose; V1, visit 1; V3, visit 3; V5, visit 5; CMM, cardiometabolic multimorbidity.

### Incidence rate and survival curve analyses

The incidence rate of CMM per 1,000 person-years was 15.31 in ELSA and 12.87 in HRS. According to quartiles of cumulative HbA1c/HDL-C ratio and its value at visit 1, the incidence rate increased progressively along with higher quartiles, with the highest rate observed in the top quartile in both ELSA and HRS, while the Q2 group of cumulative HbA1c/HDL-C ratio showed the lowest CMM risk in ELSA. Based on the trajectory of the HbA1c/HDL-C ratio, a higher incidence rate of CMM was observed in the persistently high group than in the persistently low group among both ELSA and HRS (Table 2).

**Table 2:** Incidence rate of CMM.

Exposure	Level	ELSA			HRS		
		CMM events (*n*)	Participants (*n*)	Incidence rate (95% CI)*	CMM events (*n*)	Participants (*n*)	Incidence rate (95% CI)^*^
Total		303	1918	15.31 (13.64, 17.14)	283	3,203	12.87 (11.42, 14.46)
Cumulative HbA1c/HDL-C ratio quartile	Q1	67	480	13.37 (10.36, 16.98)	50	801	9.07 (6.73, 11.96)
	Q2	51	479	9.94 (7.40, 13.07)	52	801	9.19 (6.86, 12.05)
	Q3	84	479	17.22 (13.74, 21.32)	82	801	14.84 (11.80, 18.42)
	Q4	101	480	21.19 (17.26, 25.74)	99	800	18.72 (15.22, 22.80)
HbA1c/HDL-C ratio quartile at V1	Q1	62	497	11.83 (9.07, 15.17)	44	801	7.76 (5.64, 10.41)
	Q2	72	489	14.21 (11.12, 17.90)	69	801	12.43 (9.67, 15.73)
	Q3	69	466	14.21 (11.06, 17.98)	78	800	14.27 (11.28, 17.81)
	Q4	100	466	21.62 (17.59, 26.29)	92	801	17.37 (14.00, 21.31)
HbA1c/HDL-C ratio trajectory	Persistently low trajectory	267	1783	14.43 (12.75, 16.27)	231	2,907	11.47 (10.04, 13.05)
	Persistently high trajectory	36	135	27.98 (19.59, 38.73)	52	296	28.11 (21.00, 36.87)

*Per 1,000 person-years. CMM, cardiometabolic multimorbidity; V1, visit 1.

**Figure 3:**
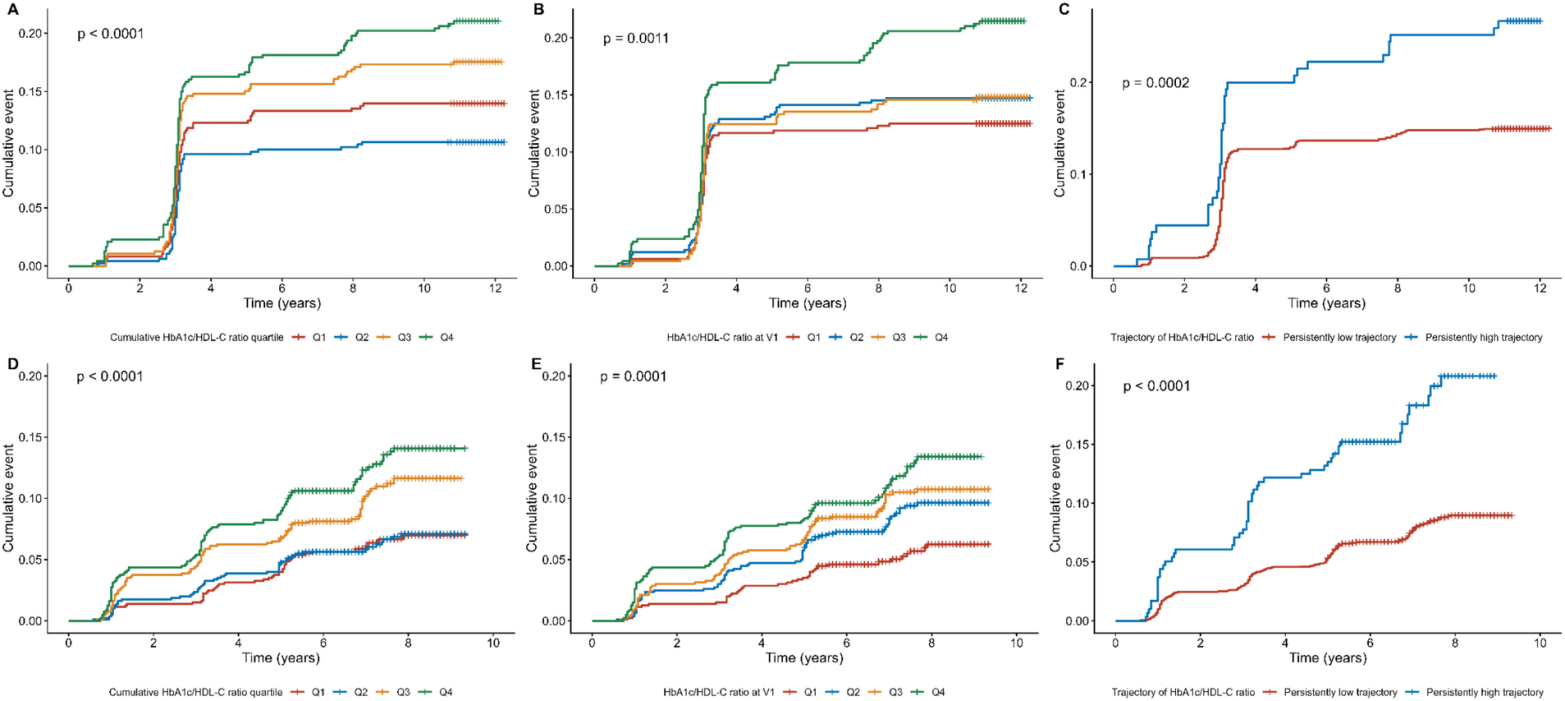
Kaplan–Meier curve analysis depicting the cumulative incidence of CMM across the quartiles of cumulative HbA1c/HDL-C ratio, its value at visit 1, and its trajectory in ELSA **(A–C)** and HRS **(D–F)**. CMM, cardiometabolic multimorbidity; V1, visit 1.

Kaplan–Meier survival analysis illustrated a gradual increase in cumulative CMM events from Q1 to Q4 groups based on cumulative HbA1c/HDL-C ratio and its value at visit 1, with statistically significant differences in both ELSA and HRS (all log-rank test *p* < 0.05, Figures 3A,B,D,E). In addition, the Kaplan–Meier curve revealed a significant elevation in cumulative incidence of CMM in the persistently high HbA1c/HDL-C ratio trajectory compared with the persistently low trajectory (all log-rank test *p* < 0.001, Figures 3C,F).

### Associations between HbA1c/HDL-C ratio and CMM

Three Cox proportional hazards models were developed to evaluate the association between HbA1c/HDL-C ratio and CMM risk (Tables 3, 4). After adjusting for all covariates, per 1-SD increase in cumulative HbA1c/HDL-C ratio was significantly related to higher CMM risk (pooled HR: 1.33, 95% CI: 1.19–1.47, *p* < 0.001; HR: 1.25, 95% CI: 1.10–1.43, *p* < 0.001 in ELSA; HR: 1.39, 95% CI: 1.23–1.58, *p* < 0.001 in HRS). Compared with the cumulative HbA1c/HDL-C ratio Q1, a 50% higher risk of CMM was observed in Q4 (pooled HR: 1.50, 95% CI: 1.15–1.95, *p* = 0.003), and the risk increased with higher quartiles (*p* for trend <0.05 in pooled analysis, ELSA, and HRS). For HbA1c/HDL-C ratio at V1, each 1-SD increase was associated with elevated risk in model 3 (pooled HR: 1.24, 95% CI: 1.07–1.43, *p* = 0.004), with the risk rising across quartiles (pooled *p* for trend<0.001). Based on trajectory of HbA1c/HDL-C ratio, persistently high HbA1c/HDL-C ratio group showed significantly elevated risk compared with persistently low group after adjustment for all confounding factors (pooled HR: 2.00, 95% CI: 1.45–2.74, *p* < 0.001; HR: 1.68, 95% CI: 1.16–2.42, *p* = 0.006 in ELSA; HR: 2.32, 95% CI: 1.68–3.21, *p* < 0.001 in HRS).

**Table 3:** Multivariate Cox regression of the relationships between HbA1c/HDL-C ratio and the risk of incident CMM in ELSA and HRS.

Characteristics	ELSA						HRS					
	Model 1		Model 2		Model 3		Model 1		Model 2		Model 3	
	HR (95% CI)	*p*-value	HR (95% CI)	*p*-value	HR (95% CI)	*p*-value	HR (95% CI)	*p*-value	HR (95% CI)	*p*-value	HR (95% CI)	*p*-value
Cumulative HbA1c/HDL-C ratio												
Per 1 SD	1.32 (1.18, 1.48)	<0.001	1.29 (1.14, 1.46)	<0.001	1.25 (1.10, 1.43)	<0.001	1.44 (1.29, 1.60)	<0.001	1.37 (1.22, 1.53)	<0.001	1.39 (1.23, 1.58)	<0.001
Quartile												
Q1	Ref		Ref		Ref		Ref		Ref		Ref	
Q2	0.75 (0.52, 1.07)	0.115	0.75 (0.52, 1.08)	0.122	0.73 (0.51, 1.06)	0.099	0.99 (0.67, 1.46)	0.954	0.90 (0.61, 1.33)	0.597	0.88 (0.59, 1.30)	0.518
Q3	1.30 (0.93, 1.80)	0.122	1.26 (0.89, 1.77)	0.187	1.20 (0.85, 1.69)	0.294	1.61 (1.13, 2.30)	0.009	1.38 (0.96, 1.99)	0.083	1.33 (0.92, 1.93)	0.126
Q4	1.58 (1.14, 2.18)	0.006	1.48 (1.04, 2.10)	0.028	1.35 (0.93, 1.95)	0.110	2.05 (1.45, 2.90)	<0.001	1.68 (1.16, 2.43)	0.006	1.67 (1.14, 2.44)	0.008
*p* for trend		<0.001		0.003		0.022		<0.001		<0.001		<0.001
HbA1c/HDL-C ratio at V1												
Per 1 SD	1.23 (1.10, 1.38)	<0.001	1.20 (1.07, 1.36)	0.002	1.15 (1.00, 1.31)	0.053	1.36 (1.22, 1.52)	<0.001	1.28 (1.14, 1.44)	<0.001	1.33 (1.17, 1.50)	<0.001
Quartile												
Q1	Ref		Ref		Ref		Ref		Ref		Ref	
Q2	1.19 (0.84, 1.67)	0.323	1.16 (0.82, 1.63)	0.404	1.14 (0.81, 1.61)	0.460	1.56 (1.07, 2.28)	0.022	1.40 (0.95, 2.05)	0.087	1.37 (0.93, 2.01)	0.108
Q3	1.20 (0.85, 1.71)	0.299	1.15 (0.80, 1.65)	0.449	1.10 (0.76, 1.58)	0.620	1.80 (1.24, 2.62)	0.002	1.54 (1.05, 2.25)	0.029	1.47 (1.00, 2.17)	0.051
Q4	1.79 (1.29, 2.50)	<0.001	1.67 (1.17, 2.37)	0.005	1.48 (1.00, 2.18)	0.049	2.20 (1.52, 3.19)	<0.001	1.79 (1.21, 2.63)	0.003	1.82 (1.21, 2.71)	0.004
*p* for trend		<0.001		0.006		0.072		<0.001		0.004		0.004
Trajectory of HbA1c/HDL-C ratio												
Persistently low trajectory	Ref		Ref		Ref		Ref		Ref		Ref	
Persistently high trajectory	1.86 (1.31, 2.66)	<0.001	1.72 (1.20, 2.48)	0.003	1.68 (1.16, 2.42)	0.006	2.44 (1.80, 3.31)	<0.001	2.19 (1.61, 2.97)	<0.001	2.32 (1.68, 3.21)	<0.001

Model 1 included adjustment for age and sex. Model 2 was further adjusted for marital status, educational level, smoking status, drinking status, and WC. Model 3 additionally controlled for dyslipidemia, hypertension, cancer, TC, and CRP. CMM, cardiometabolic multimorbidity; V1, visit 1.

**Table 4:** Pooled analysis of associations between HbA1c/HDL-C ratio and CMM risk in different models.

Pooled analysis												
Characteristics	Model 1				Model 2				Model 3			
	HR (95% CI)	*p*-value*	I^2^ (%)	*p*-value^#^	HR (95% CI)	*p*-value*	I^2^ (%)	*p*-value^#^	HR (95% CI)	*p*-value*	I^2^ (%)	*p*-value^#^
Cumulative HbA1c/HDL-C ratio												
Per 1 SD	1.38 (1.27, 1.50)	<0.001	15.6	0.276	1.33 (1.22, 1.45)	<0.001	0.0	0.529	1.33 (1.19, 1.47)	<0.001	27.0	0.242
Quartile												
Q1	Ref				Ref				Ref			
Q2	0.85 (0.65, 1.12)	0.252	6.5	0.301	0.82 (0.62, 1.07)	0.136	0.0	0.501	0.80 (0.61, 1.04)	0.100	0.0	0.511
Q3	1.43 (1.12, 1.82)	0.004	0.0	0.384	1.31 (1.02, 1.69)	0.032	0.0	0.708	1.26 (0.98, 1.62)	0.070	0.0	0.683
Q4	1.78 (1.38, 2.31)	<0.001	14.2	0.280	1.57 (1.22, 2.03)	<0.001	0.0	0.627	1.50 (1.15, 1.95)	0.003	0.0	0.429
*p* for trend		<0.001				<0.001				<0.001		
HbA1c/HDL-C ratio at V1												
Per 1 SD	1.30 (1.18, 1.43)	<0.001	37.4	0.206	1.24 (1.14, 1.35)	<0.001	0.0	0.456	1.24 (1.07, 1.43)	0.004	57.8	0.124
Quartile												
Q1	Ref				Ref				Ref			
Q2	1.34 (1.03, 1.75)	0.029	8.1	0.297	1.26 (0.97, 1.63)	0.078	0.0	0.473	1.24 (0.96, 1.60)	0.105	0.0	0.484
Q3	1.46 (0.99, 2.17)	0.058	57.9	0.123	1.32 (0.99, 1.75)	0.057	14.2	0.280	1.26 (0.95, 1.68)	0.113	13.7	0.282
Q4	1.97 (1.54, 2.52)	<0.001	0.0	0.420	1.72 (1.33, 2.23)	<0.001	0.0	0.792	1.63 (1.23, 2.16)	<0.001	0.0	0.471
*p* for trend		<0.001				<0.001				<0.001		
Trajectory of HbA1c/HDL-C ratio												
Persistently low trajectory	Ref				Ref				Ref			
Persistently high trajectory	2.17 (1.67, 2.82)	<0.001	22.5	0.256	1.98 (1.56, 2.50)	<0.001	0.0	0.330	2.00 (1.45, 2.74)	<0.001	41.5	0.191

Model 1 included adjustment for age and sex. Model 2 was further adjusted for marital status, educational level, smoking status, drinking status, and WC. Model 3 additionally controlled for dyslipidemia, hypertension, cancer, TC, and CRP. *p*-value* for the pooled HR 95%CI. *p*-value^#^ for the heterogeneity. CMM, cardiometabolic multimorbidity; V1, visit 1.

**Figure 4:**
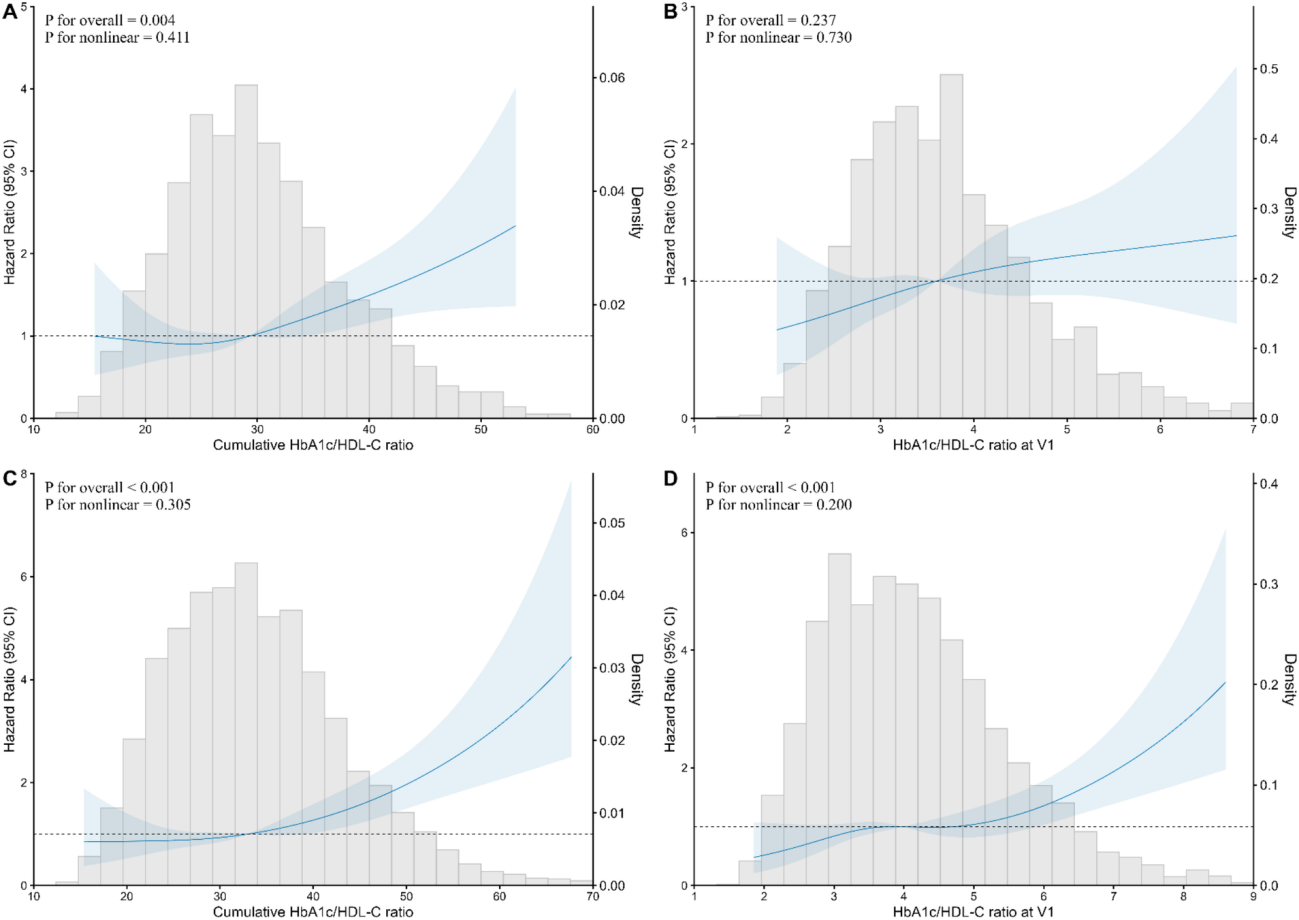
Restricted cubic spline curves depicting the association between CMM and cumulative HbA1c/HDL-C ratio, and its value at visit 1 in ELSA **(A,B)** and HRS **(C,D)** after adjustment for covariates. The thick central line represents the estimated adjusted hazard ratio, and the shaded area indicates the 95% confidence interval. The model was adjusted for age, sex, marital status, educational level, smoking status, drinking status, WC, dyslipidemia, hypertension, cancer, TC, and CRP. CMM, cardiometabolic multimorbidity; V1, visit 1.

Multivariable-adjusted restricted cubic spline regression model indicated a significantly positive, linear relationship between occurrence of CMM events and cumulative HbA1c/HDL-C ratio in ELSA and HRS, as well as HbA1c/HDL-C ratio at visit 1 in HRS (all *p* for overall <0.05 and *p* for non-linear >0.05) (Figure 4).

### Predictive value and mediation analysis of HbA1c/HDL-C ratio for CMM

**Figure 5:**
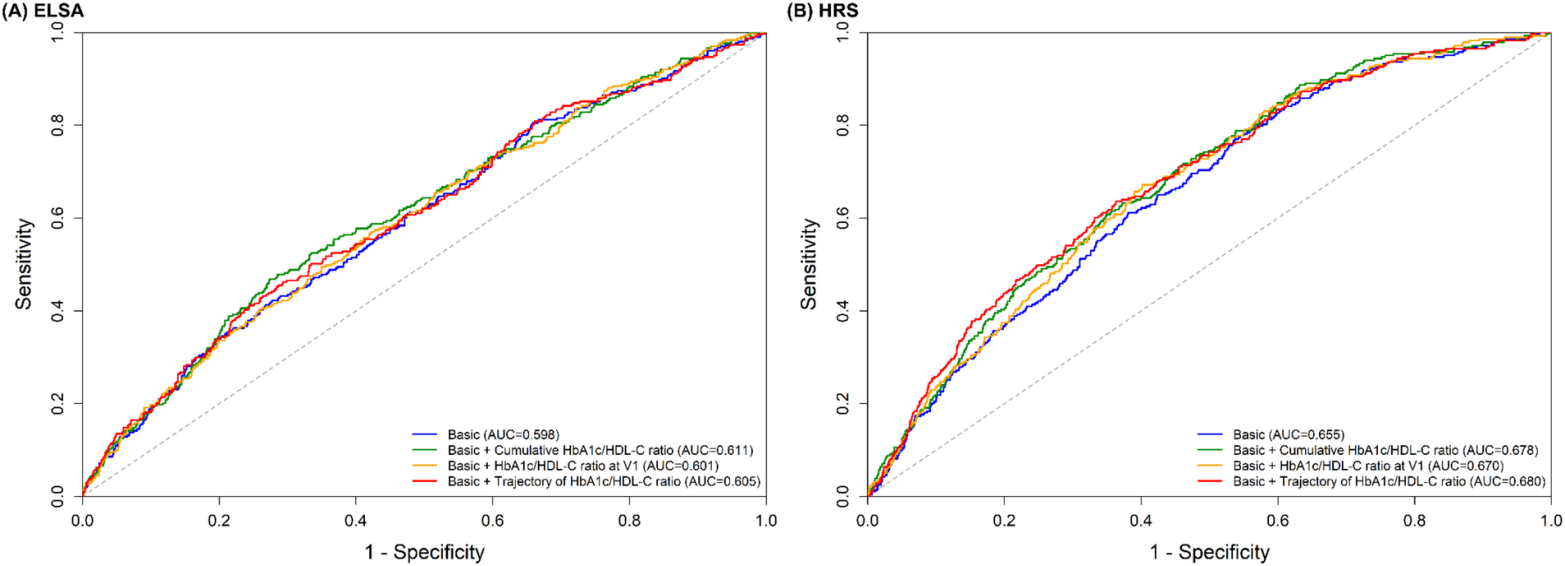
Receiver operating characteristic curves for HbA1c/HDL-C ratio predicting CMM incidence in ELSA **(A)** and HRS **(B)**. Basic model included age, sex, marital status, educational level, smoking status, drinking status, WC, dyslipidemia, hypertension, cancer, TC, and CRP. CMM, cardiometabolic multimorbidity; V1, visit 1.

The predictive performance of the HbA1c/HDL-C ratio for incident CMM was assessed by ROC curve analysis, NRI, and IDI (Figure 5, Supplementary Table S14). Adding the cumulative HbA1c/HDL-C ratio into the basic model (model 3) significantly improved the predictive capacity in the ELSA (IDI: 0.006, 95% CI: 0.001, 0.015). In the HRS, the combination of basic model and cumulative or trajectory of HbA1c/HDL-C ratio increased the AUC (0.678, 95% CI: 0.648–0.709 and 0.680, 95% CI: 0.649–0.712, respectively, all *p* < 0.05 by DeLong’s test). Cumulative HbA1c/HDL-C ratio, its value at visit 1, and its trajectory were effective predictors (NRI > 0 and IDI > 0 with all *p* < 0.05), with the greatest enhancement observed in cumulative HbA1c/HDL-C ratio (NRI: 0.269, 95% CI: 0.111–0.370; IDI: 0.012, 95% CI: 0.004–0.027).

After adjusting for covariates, BMI was identified as a mediator between the trajectory of HbA1c/HDL-C ratio and CMM risk in HRS, with the total effect of −50.9182, ADE of −50.0776, ACME of −1.8463, and a relatively low proportion mediated of 3.33% (*p* = 0.034) (Supplementary Table S15).

### Subgroup analyses

**Figure 6:**
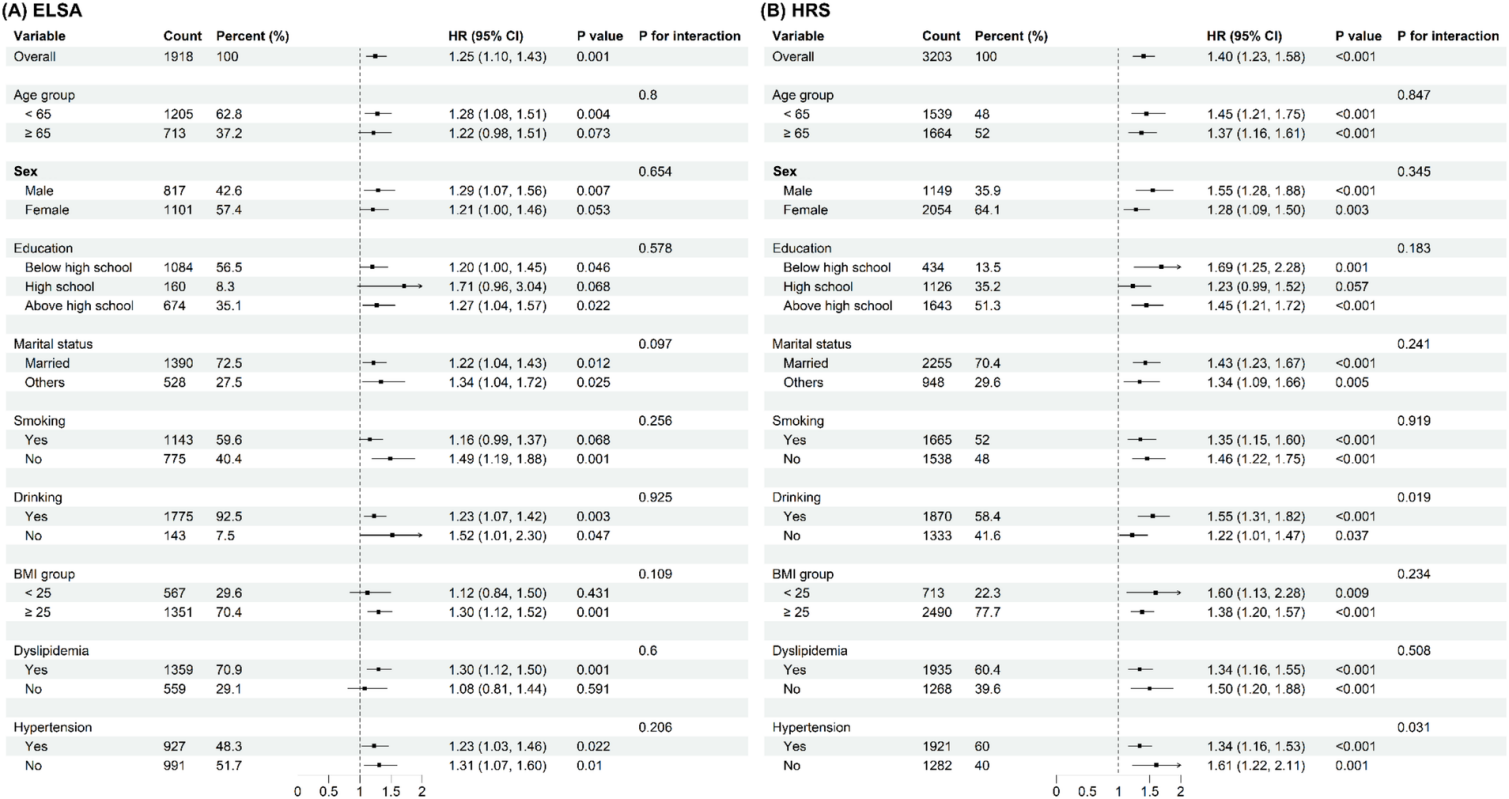
Subgroup and interaction analyses of the associations between cumulative HbA1c/HDL-C ratio and CMM risk in ELSA **(A)** and HRS **(B)**.

Figure 6 and Supplementary Figures S2, S3 present the associations of HbA1c/HDL-C ratio and CMM risk stratified by age, gender, education levels, marital status, smoking status, drinking status, BMI, hypertension, and dyslipidemia. The stratified analyses indicated that significant interaction effects were observed between cumulative HbA1c/HDL-C ratio and drinking status, hypertension, as well as trajectory of HbA1c/HDL-C ratio and dyslipidemia in HRS. No significant interactions were observed in the other subgroups (all *p* for interaction > 0.05).

### Sensitivity analyses

To evaluate the robustness of our findings, several sensitivity analyses were performed. Generally, the positive associations between HbA1c/HDL-C ratio and incident CMM observed in the primary analysis remained largely consistent when using logistic regression models (Supplementary Tables S16, S17), excluding participants with missing covariates (Supplementary Tables S18, S19), excluding those receiving medications for certain conditions (heart disease, stroke, diabetes, hypertension, or dyslipidemia) at baseline (Supplementary Tables S20, S21), or excluding participants with less than 2 years of follow-up (Supplementary Tables S22, S23). Based on fully adjusted models, the E-value suggested that this association was relatively robust to unmeasured confounding factors (Supplementary Table S24).

## Discussion

To the best of our knowledge, this is the first study focusing on the association between HbA1c/HDL-C ratio and CMM in the ELSA and HRS. After adjustment for all covariates, a higher HbA1c/HDL-C ratio (cumulative, visit 1 value, and trajectory) significantly increases the risk of incident CMM. The predictive performance of the basic model is ameliorated by adding the HbA1c/HDL-C ratio. Therefore, consistent detection of the HbA1c/HDL-C ratio may be noteworthy for early prediction and timely intervention of CMM.

Previous studies have shown that the TG/HDL-C ratio serves as a simple, inexpensive, and non-invasive marker for cardiovascular risk prediction, especially among individuals with prediabetes or insulin resistance (32). Similarly, the triglyceride-glucose index has been extensively applied as an indicator of insulin resistance and atherosclerotic risk, with the added advantage of easy calculation, which makes it well suited for epidemiological studies and community-based screening (33). However, these indicators mainly capture specific metabolic components or transient metabolic disturbances and therefore do not adequately reflect the intricate crosstalk between glucose and lipid homeostasis. In contrast, the HbA1c/HDL-C ratio integrates chronic glycemic burden, as indicated by HbA1c, with the diverse protective functions of HDL-C, allowing it to more effectively represent the overall cumulative impact of glucolipid metabolic derangement.

The components of the HbA1c/HDL-C ratio are highly related to the components of CMM, i.e., stroke, diabetes, and heart disease. HbA1c level ≥ 6.5% was included as a diagnostic criterion for diabetes by the American Diabetes Association in 2010 (34). In addition, a comprehensive meta-analysis showed that higher HbA1c was associated with a greater likelihood of incident stroke among patients with diabetes (35). Cohort studies have further demonstrated that HbA1c is related to the prognosis of individuals with coronary heart disease and that its concentration may serve as a predictor of cardiovascular disease risk, thereby facilitating the identification of high-risk populations for targeted prevention and intervention (36, 37). A prospective study including 4,585 individuals with diabetes from the UK showed that a 1% reduction in HbA1c was associated with a 21% lower risk of diabetes-associated endpoints, a 14% lower risk of myocardial infarction, and a 37% lower risk of microvascular complications (38). HDL particles are well recognized for their anti-atherosclerotic effects (39). In this context, the Framingham Study demonstrated an inverse association between HDL-C levels and coronary heart disease risk, suggesting that therapeutic elevation of HDL may contribute to the prevention of coronary heart disease (40). Evidence from the UK Biobank further indicated that HDL may lose part of its atheroprotective function in individuals with diabetes (41), highlighting a specific interaction between HDL and diabetes. Similarly, HDL-C concentrations have also been reported to be inversely associated with the risk of stroke (42, 43). Higher HDL-C concentrations have been persistently associated with reduced likelihood of cardiovascular events, even in subjects with LDL-C lower than 70 mg/dL (44, 45).

Previous studies have determined the relationships between CMM and multiple glucolipid metabolic indices, such as visceral adiposity index, triglyceride glucose, estimated glucose disposal rate, metabolic score for insulin resistance, cholesterol, high-density lipoprotein, glucose index, and atherogenic index of plasma (10, 26, 46–48). Wu et al. and Zheng et al. both conducted a cross-sectional study and demonstrated a positive relationship between HbA1c/HDL-C ratio and non-alcoholic fatty liver disease among the total or non-diabetic population based on the 2017–2020 NHANES in the US (49, 50). Huang et al. (22) reported that higher HbA1c/HDL-C ratio and cumulative mean HbA1c/HDL-C ratio were both related to elevated stroke incidence by performing a cross-sectional study and cohort research based on data from the 2011–2018 China Health and Retirement Longitudinal Study (CHARLS). Dong et al. (21) reported that participants with both an HbA1c/HDL-C ratio ≥ median value and depressive status had significantly elevated incident CMM compared with those with an HbA1c/HDL-C ratio < median value and non-depression in the CHARLS cohort. A longitudinal study included 4,225 Chinese aged 45 years and older from CHARLS and showed a positive linear association between cumulative mean HbA1c/HDL-C ratio and CMM event (51).

Our study reveals mostly consistent findings, indicating that elevated incident CMM is significantly linearly associated with higher cumulative HbA1c/HDL-C ratio and its value at visit 1 in ELSA, HRS, and pooled analysis, except for HbA1c/HDL-C ratio at visit 1. Such disparity may result from the relatively small sample size in the ELSA cohort and the fact that subjects with CMM occurrence prior to or during the 10-year baseline surveys are excluded, retaining individuals with relatively superior health profiles. Additions of cumulative HbA1c/HDL-C ratio, its value at visit 1, and its trajectory reveal noteworthy incremental predictive value. The cumulative HbA1c/HDL-C ratio appears to be the optimal predictor as it significantly improves the AUC, NRI, and IDI in the ELSA and HRS. The mediation analysis shows that the association between the trajectory of HbA1c/HDL-C ratio and CMM was partially mediated by BMI, which underscores the essentiality of BMI monitoring apart from HbA1c/HDL-C ratio detection in subjects with high risk of incident CMM. Subgroup analyses indicate that the positive relationship between the cumulative HbA1c/HDL-C ratio and CMM risk is more significant in alcohol drinkers in the HRS. The association of alcohol consumption and diabetes, stroke, and heart disease is complex. A comprehensive review reports that moderate alcohol intake would lead to beneficial effects for cardiovascular disease and diabetes, whereas alcohol abuse results in opposite outcomes (52).

The potential mechanisms can be interpreted from multiple aspects. To begin with, a higher HbA1c/HDL-C ratio may serve as an indicator of metabolic dysregulation. The coexistence of elevated HbA1c and reduced HDL-C suggests concurrent disturbances in glucose and lipid homeostasis. Increased HbA1c reflects suboptimal glycemic control, while sustained hyperglycemia can suppress endothelial nitric oxide (NO) production, thereby contributing to reduced vasodilatory capacity, greater vascular resistance, endothelial injury, and persistent low-grade inflammation (53–56). Concurrently, decreased HDL-C diminishes its anti-atherogenic functions, facilitating cholesterol deposition within the arterial walls and accelerating atherosclerotic processes (57). Moreover, inflammatory and oxidative stress pathways exacerbate cardiometabolic risk (58, 59). Hyperglycemia promotes the production of pro-inflammatory mediators, including IL-6 and TNF-*α*, thereby driving a state of chronic systemic inflammation. This inflammatory milieu can further worsen insulin resistance, weaken the protective functions of HDL-C, and increase the risk of CMM (60). Moreover, elevated glucose concentrations intensify oxidative stress, facilitating LDL-C oxidation and the formation of oxidized LDL (ox-LDL), which in turn accelerates the development of atherosclerosis. Low HDL-C fails to efficiently remove oxidative byproducts, further aggravating vascular injury (61, 62). Insulin resistance also plays a key role in lipid dysregulation. Hyperinsulinemia suppresses lipolysis while increasing very-low-density lipoprotein (VLDL) and LDL concentrations and decreasing HDL-C levels, thereby contributing to atherosclerotic progression (63). Accordingly, an elevated HbA1c/HDL-C ratio may indicate the coexistence of impaired glucose regulation and lipid metabolic abnormalities. Through multiple interconnected mechanisms, these metabolic derangements contribute to the development of atherosclerosis, cardiovascular disease, and type 2 diabetes. In contrast, a lower HbA1c/HDL-C ratio is more likely to reflect a relatively stable metabolic state and a lower risk of CMM, whereas a higher ratio suggests more pronounced metabolic dysregulation, stronger inflammatory activation, and more severe vascular damage, thereby increasing vulnerability to CMM.

Our study has several advantages. First, the HbA1c/HDL-C ratio was comprehensively examined using cumulative values, visit 1 values, and trajectory patterns, which strengthened the robustness and potential clinical applicability of the findings. The repeated measurements rather than single timepoint assessment allow the metric evaluation to take temporal variations into account, offering a multidimensional perspective. Second, the 20-year prospective longitudinal design, based on two large nationally representative cohorts, substantially enhanced the reliability of the results. Finally, as a simple, inexpensive, and readily accessible biomarker, the HbA1c/HDL-C ratio may facilitate the identification of individuals at high risk and provide useful information for early intervention and clinical management.

This study has several limitations to declare: First, the study population consisted of middle-aged and older adults from two national cohorts, which may limit the applicability of the findings to younger individuals, other ethnic populations, and different countries. Additional studies, including international and multiethnic investigations as well as analyses involving individuals aged 50 years or younger, are needed to further confirm these associations. Second, despite adjustment for multiple covariates, the possibility of residual confounding due to unmeasured or inadequately controlled factors cannot be excluded. Third, CMM events were identified on the basis of self-reported physician diagnoses, which may have introduced information bias or misclassification. Fourth, as the exact timing of CMM onset was unavailable, the Cox proportional hazards models could only estimate risk according to follow-up duration, which may have influenced the precision of the findings. Finally, given the observational nature of the study, causal relationships cannot be established.

Future research should proceed in three complementary directions. First, mechanistic studies are needed to clarify how an elevated HbA1c/HDL-C ratio contributes to the development of CMM. Given that BMI explained only a small proportion of the observed association, additional pathways beyond adiposity likely play important roles, including insulin resistance, atherogenic dyslipidemia, chronic low-grade inflammation, endothelial dysfunction, and oxidative stress. Second, randomized controlled trials or pragmatic intervention studies should evaluate whether reducing the HbA1c/HDL-C ratio can lower CMM risk, particularly among older adults with persistently high trajectories. Potential strategies may include structured lifestyle modification, weight management, dietary optimization, and pharmacological interventions targeting glycemic control and lipid metabolism. Third, future studies are warranted to investigate whether combining the HbA1c/HDL-C ratio with other metabolic and inflammatory biomarkers can further improve risk stratification and predictive performance for CMM. Such integrative approaches may provide a more comprehensive assessment of cardiometabolic risk and facilitate the development of personalized prevention strategies.

From a clinical perspective, the HbA1c/HDL-C ratio may serve as a simple and readily available marker for identifying middle-aged and older adults at elevated risk of CMM. Individuals with persistently high HbA1c/HDL-C ratio trajectories may benefit from closer metabolic monitoring and early lifestyle or risk-factor management to potentially reduce the burden of cardiometabolic diseases.

## Conclusion

In summary, positive linear associations between cumulative HbA1c/HDL-C ratio, its value at visit 1, and risk of incident CMM are observed among adults aged 50 years and older in the UK and the US. Participants with a consistently high HbA1c/HDL-C ratio trajectory had a higher risk of incident CMM. In addition, the addition of the HbA1c/HDL-C ratio significantly enhances the predictive performance for CMM. Our findings offer preliminary evidence that may contribute to the development of future prevention strategies, and additional investigations are required to further confirm and generalize these results.

## Data Availability

Publicly available datasets were analyzed in this study. This data can be found at: ELSA: https://ukdataservice.ac.uk/about/; HRS: https://hrs.isr.umich.edu.
