# Supplementary material for: The association between HbA1c/HDL-C ratio and cardiometabolic multimorbidity among middle-aged and older adults: data from two national prospective cohorts in the United Kingdom and United States
Source: Front Nutr. 2026 Jun 29;13:1849520. doi: 10.3389/fnut.2026.1849520 (PMC13357312; doi:10.3389/fnut.2026.1849520)
Supplement: Supplementary file 1 [file Supplementary_file_1.DOCX]

Supplementary materials

Supplementary methods: detailed information of ELSA and HRS

The English Longitudinal Study of Ageing (ELSA)

The English Longitudinal Study of Ageing (ELSA) is a longitudinal investigation of ageing and quality of life among adults aged 50 years and above in the United Kingdom. In wave 1, conducted between March 2002 and March 2003, the original cohort comprised 11,050 respondents, and follow-up assessments have been carried out every two years thereafter. The survey gathers detailed data on demographics, income, assets, health, cognitive function, family structure and social relationships, health care use and related costs, housing, employment status and occupational history, expectations, and insurance coverage. Information is obtained using computer-assisted personal interviews together with self-administered questionnaires. Starting from wave 2, eligible participants were invited to receive a nurse visit every four years, conducted by trained nursing professionals. During these visits, biological specimens, physical examination findings, and performance-based measures were collected for further analysis. Ethical approval for all waves of ELSA was provided by the National Health Service Research Ethics Committees under the National Research and Ethics Service (NRES).

The Health and Retirement Study (HRS)

The Health and Retirement Study (HRS) is an ongoing, prospective, nationally representative survey involving over 42,000 adults aged 50 years and older in the United States. Its baseline wave was launched in 1992, and participants have subsequently been followed at 2-year intervals. The survey collects extensive information on demographic characteristics, health status, cognitive function, family composition, housing conditions, employment and pension status, health care utilization and expenditures, expectations, assets, and income. Beginning in 2006 (wave 8), the scope of data collection was broadened to incorporate biomarker assessments and more detailed psychological measures. At that wave, one-half of the sample was randomly selected in advance to undergo an enhanced face-to-face interview (EFTF), which included physical performance assessments, anthropometric evaluations, collection of blood and saliva specimens, and a self-completed questionnaire addressing psychosocial domains. These data contribute to the validation and enrichment of self-reported health information and may also identify health-related characteristics that are either unknown to the participants themselves or not captured through self-report measures. Ethical approval for the HRS was granted by the Institutional Review Board of the University of Michigan.

Table S1 Distribution of variables with missing data in ELSA

| **Variables** | **Number of missing** | **Missing proportion** |
| --- | --- | --- |
| Education | 165 | 8.60% |
| Drinking | 107 | 5.58% |
| BMI | 55 | 2.87% |
| Waist circumference | 24 | 1.25% |
| Height | 36 | 1.88% |
| Weight | 36 | 1.88% |
| SBP | 15 | 0.78% |
| DBP | 15 | 0.78% |
| Hypertension | 8 | 0.42% |
| LDL-C | 48 | 2.50% |
| FPG | 590 | 30.76% |
| CRP | 1 | 0.05% |

Table S2 Distribution of variables with missing data in HRS

| **Variables** | **Number of missing** | **Missing proportion** |
| --- | --- | --- |
| Marital status | 1 | 0.03% |
| Residence | 3 | 0.09% |
| Smoking | 21 | 0.66% |
| Drinking | 1 | 0.03% |
| BMI | 120 | 3.75% |
| Waist circumference | 60 | 1.87% |
| Height | 39 | 1.22% |
| Weight | 112 | 3.50% |
| SBP | 58 | 1.81% |
| DBP | 58 | 1.81% |
| Hypertension | 28 | 0.87% |
| Hypertension treatment | 3 | 0.09% |
| Diabetes treatment | 3 | 0.09% |
| Heart disease | 1 | 0.03% |
| TC | 1 | 0.03% |
| CRP | 39 | 1.22% |

Table S3 Fit indices of GBTM model on HbA1c/HDL-C ratio trajectories in ELSA

| Trajectory | AIC | BIC | aBIC | Entropy | APP | Class (%) |
| --- | --- | --- | --- | --- | --- | --- |
| One trajectory | 13467.9 | 13490.14 | 13477.43 | NA | 1 | 100 |
| Two trajectory | 13074.42 | 13113.33 | 13091.09 | 0.873 | 0.97/0.85 | 92.96/7.04 |
| Three trajectory | 12866.71 | 12922.3 | 12890.53 | 0.921 | 0.97/0.88/0.86 | 91.81/2.24/5.94 |
| Four trajectory | 12775.11 | 12847.37 | 12806.07 | 0.828 | 0.74/0.85/0.93/0.84 | 11.11/2.4/82.12/4.38 |
| Five trajectory | 12737.48 | 12826.43 | 12775.59 | 0.829 | 0.89/0.73/0.72/0.93/0.84 | 1.77/3.08/12.36/79.98/2.82 |

Abbreviations: AIC, akaike information criterion; BIC, bayesian information criterion; aBIC, adjusted bayesian information criterion; APP, average posterior probability.

Table S4 Fit indices of GBTM model on HbA1c/HDL-C ratio trajectories in HRS

| Trajectory | AIC | BIC | aBIC | Entropy | APP | Class (%) |
| --- | --- | --- | --- | --- | --- | --- |
| One trajectory | 27980.06 | 28004.35 | 27991.64 | NA | 1 | 100 |
| Two trajectory | 27806.97 | 27849.47 | 27827.23 | 0.755 | 0.95/0.78 | 90.76/9.24 |
| Three trajectory | 27512.26 | 27572.98 | 27541.21 | 0.804 | 0.94/0.81/0.79 | 85.36/4.5/10.15 |
| Four trajectory | 27518.26 | 27597.2 | 27555.89 | 0.434 | NA/0.52/0.76/0.71 | 0/82.08/5.12/12.8 |
| Five trajectory | 27524.26 | 27621.41 | 27570.57 | 0.303 | 0.34/NA/NA/0.7/0.65 | 79.24/0/0/5.9/14.86 |

Abbreviations: AIC, akaike information criterion; BIC, bayesian information criterion; aBIC, adjusted bayesian information criterion; APP, average posterior probability.

Table S5 Collinearity Statistics for cumulative HbA1c/HDL-C ratio in ELSA

| Characteristic | GVIF | Df | GVIF^(1/(2*Df)) |
| --- | --- | --- | --- |
| Cumulative HbA1c/HDL-C ratio (z-score) | 1.566 | 1 | 1.251 |
| Age | 1.103 | 1 | 1.05 |
| Gender | 1.421 | 1 | 1.192 |
| Education | 1.082 | 2 | 1.02 |
| Marital status | 1.094 | 1 | 1.046 |
| Smoking | 1.043 | 1 | 1.021 |
| Drinking | 1.056 | 1 | 1.028 |
| Waist circumference | 1.442 | 1 | 1.201 |
| Dyslipidemia | 1.357 | 1 | 1.165 |
| Hypertension | 1.131 | 1 | 1.064 |
| Cancer | 1.008 | 1 | 1.004 |
| TC | 1.427 | 1 | 1.194 |
| CRP | 1.045 | 1 | 1.022 |

Table S6 Collinearity Statistics for cumulative HbA1c/HDL-C ratio in HRS

| Characteristic | GVIF | Df | GVIF^(1/(2*Df)) |
| --- | --- | --- | --- |
| Cumulative HbA1c/HDL-C ratio (z-score) | 1.27 | 1 | 1.127 |
| Age | 1.123 | 1 | 1.06 |
| Gender | 1.247 | 1 | 1.117 |
| Education | 1.119 | 2 | 1.028 |
| Marital status | 1.084 | 1 | 1.041 |
| Smoking | 1.068 | 1 | 1.033 |
| Drinking | 1.14 | 1 | 1.068 |
| Waist circumference | 1.226 | 1 | 1.107 |
| Dyslipidemia | 1.118 | 1 | 1.057 |
| Hypertension | 1.074 | 1 | 1.036 |
| Cancer | 1.03 | 1 | 1.015 |
| TC | 1.147 | 1 | 1.071 |
| CRP | 1.093 | 1 | 1.045 |

Table S7 Collinearity Statistics for HbA1c/HDL-C ratio at V1 in ELSA

| Characteristic | GVIF | Df | GVIF^(1/(2*Df)) |
| --- | --- | --- | --- |
| HbA1c/HDL-C ratio (z-score) at V1 | 1.719 | 1 | 1.311 |
| Age | 1.103 | 1 | 1.05 |
| Gender | 1.4 | 1 | 1.183 |
| Education | 1.078 | 2 | 1.019 |
| Marital status | 1.097 | 1 | 1.047 |
| Smoking | 1.043 | 1 | 1.021 |
| Drinking | 1.05 | 1 | 1.025 |
| Waist circumference | 1.425 | 1 | 1.194 |
| Dyslipidemia | 1.396 | 1 | 1.181 |
| Hypertension | 1.132 | 1 | 1.064 |
| Cancer | 1.008 | 1 | 1.004 |
| TC | 1.662 | 1 | 1.289 |
| CRP | 1.048 | 1 | 1.024 |

Table S8 Collinearity Statistics for HbA1c/HDL-C ratio at V1 in HRS

| Characteristic | GVIF | Df | GVIF^(1/(2*Df)) |
| --- | --- | --- | --- |
| HbA1c/HDL-C ratio (z-score) at V1 | 1.337 | 1 | 1.156 |
| Age | 1.121 | 1 | 1.059 |
| Gender | 1.259 | 1 | 1.122 |
| Education | 1.122 | 2 | 1.029 |
| Marital status | 1.086 | 1 | 1.042 |
| Smoking | 1.07 | 1 | 1.034 |
| Drinking | 1.135 | 1 | 1.065 |
| Waist circumference | 1.242 | 1 | 1.114 |
| Dyslipidemia | 1.141 | 1 | 1.068 |
| Hypertension | 1.072 | 1 | 1.035 |
| Cancer | 1.029 | 1 | 1.014 |
| TC | 1.167 | 1 | 1.08 |
| CRP | 1.092 | 1 | 1.045 |

Table S9 Collinearity Statistics for the trajectory of HbA1c/HDL-C ratio in ELSA

| Characteristic | GVIF | Df | GVIF^(1/(2*Df)) |
| --- | --- | --- | --- |
| Trajectory of HbA1c/HDL-C ratio | 1.104 | 1 | 1.051 |
| Age | 1.099 | 1 | 1.048 |
| Gender | 1.389 | 1 | 1.178 |
| Education | 1.079 | 2 | 1.019 |
| Marital status | 1.097 | 1 | 1.047 |
| Smoking | 1.045 | 1 | 1.022 |
| Drinking | 1.054 | 1 | 1.027 |
| Waist circumference | 1.341 | 1 | 1.158 |
| Dyslipidemia | 1.248 | 1 | 1.117 |
| Hypertension | 1.129 | 1 | 1.063 |
| Cancer | 1.007 | 1 | 1.004 |
| TC | 1.271 | 1 | 1.128 |
| CRP | 1.044 | 1 | 1.022 |

Table S10 Collinearity Statistics for the trajectory of HbA1c/HDL-C ratio in HRS

| Characteristic | GVIF | Df | GVIF^(1/(2*Df)) |
| --- | --- | --- | --- |
| Trajectory of HbA1c/HDL-C ratio | 1.156 | 1 | 1.075 |
| Age | 1.123 | 1 | 1.06 |
| Gender | 1.231 | 1 | 1.109 |
| Education | 1.12 | 2 | 1.029 |
| Marital status | 1.08 | 1 | 1.039 |
| Smoking | 1.07 | 1 | 1.034 |
| Drinking | 1.14 | 1 | 1.068 |
| Waist circumference | 1.201 | 1 | 1.096 |
| Dyslipidemia | 1.136 | 1 | 1.066 |
| Hypertension | 1.075 | 1 | 1.037 |
| Cancer | 1.029 | 1 | 1.015 |
| TC | 1.14 | 1 | 1.068 |
| CRP | 1.09 | 1 | 1.044 |

Table S11 Patient demographics and baseline characteristics based on quartiles of HbA1c/HDL-C ratio at visit 1

| **Characteristic** | **ELSA** | | | | | | **HRS** | | | | | |
| --- | --- | --- | --- | --- | --- | --- | --- | --- | --- | --- | --- | --- |
|  | **Overall (N = 1,918)** | **Q1: 1.38-3.00 (N = 497)** | **Q2: 3.00-3.60 (N = 489)** | **Q3: 3.61-4.23 (N = 466)** | **Q4: 4.24-7.18 (N = 466)** | **P-value** | **Overall (N = 3,203)** | **Q1: 1.38-3.16 (N = 801)** | **Q2: 3.16-3.98 (N = 801)** | **Q3: 3.99-4.92 (N = 800)** | **Q4: 4.92-9.37 (N = 801)** | **P-value** |
| Age | 61.00 (57.00, 68.00) | 61.00 (56.00, 67.00) | 61.00 (57.00, 68.00) | 61.00 (56.00, 67.00) | 62.00 (57.00, 69.00) | 0.225 | 65.00 (58.00, 71.00) | 63.00 (57.00, 70.00) | 66.00 (59.00, 71.00) | 65.50 (58.00, 71.00) | 65.00 (58.00, 71.00) | <0.001 |
| Gender |  |  |  |  |  | <0.001 |  |  |  |  |  | <0.001 |
| Male | 817 (42.6%) | 107 (21.5%) | 169 (34.6%) | 239 (51.3%) | 302 (64.8%) |  | 1,149 (35.9%) | 160 (20.0%) | 239 (29.8%) | 321 (40.1%) | 429 (53.6%) |  |
| Female | 1,101 (57.4%) | 390 (78.5%) | 320 (65.4%) | 227 (48.7%) | 164 (35.2%) |  | 2,054 (64.1%) | 641 (80.0%) | 562 (70.2%) | 479 (59.9%) | 372 (46.4%) |  |
| Education |  |  |  |  |  | 0.580 |  |  |  |  |  | <0.001 |
| Below high school | 1,084 (56.5%) | 273 (54.9%) | 279 (57.1%) | 256 (54.9%) | 276 (59.2%) |  | 434 (13.5%) | 68 (8.5%) | 101 (12.6%) | 112 (14.0%) | 153 (19.1%) |  |
| High school | 160 (8.3%) | 41 (8.2%) | 35 (7.2%) | 46 (9.9%) | 38 (8.2%) |  | 1,126 (35.2%) | 262 (32.7%) | 270 (33.7%) | 295 (36.9%) | 299 (37.3%) |  |
| Above high school | 674 (35.1%) | 183 (36.8%) | 175 (35.8%) | 164 (35.2%) | 152 (32.6%) |  | 1,643 (51.3%) | 471 (58.8%) | 430 (53.7%) | 393 (49.1%) | 349 (43.6%) |  |
| Marital status |  |  |  |  |  | <0.001 |  |  |  |  |  | 0.492 |
| Married | 1,390 (72.5%) | 338 (68.0%) | 325 (66.5%) | 359 (77.0%) | 368 (79.0%) |  | 2,255 (70.4%) | 567 (70.8%) | 552 (68.9%) | 557 (69.6%) | 579 (72.3%) |  |
| Others | 528 (27.5%) | 159 (32.0%) | 164 (33.5%) | 107 (23.0%) | 98 (21.0%) |  | 948 (29.6%) | 234 (29.2%) | 249 (31.1%) | 243 (30.4%) | 222 (27.7%) |  |
| Residence |  |  |  |  |  | NA |  |  |  |  |  | 0.918 |
| Urban | NA | NA | NA | NA | NA |  | 2,141 (66.8%) | 532 (66.4%) | 530 (66.2%) | 541 (67.6%) | 538 (67.2%) |  |
| Rural | NA | NA | NA | NA | NA |  | 1,062 (33.2%) | 269 (33.6%) | 271 (33.8%) | 259 (32.4%) | 263 (32.8%) |  |
| Smoking | 1,143 (59.6%) | 268 (53.9%) | 275 (56.2%) | 293 (62.9%) | 307 (65.9%) | <0.001 | 1,665 (52.0%) | 395 (49.3%) | 429 (53.6%) | 405 (50.6%) | 436 (54.4%) | 0.132 |
| Drinking | 1,775 (92.5%) | 464 (93.4%) | 457 (93.5%) | 434 (93.1%) | 420 (90.1%) | 0.162 | 1,870 (58.4%) | 561 (70.0%) | 460 (57.4%) | 449 (56.1%) | 400 (49.9%) | <0.001 |
| BMI (kg/m^2^) | 27.52 (4.53) | 25.68 (4.02) | 27.26 (4.33) | 28.30 (4.49) | 28.97 (4.60) | <0.001 | 28.50 (25.30, 32.30) | 26.50 (23.90, 30.40) | 27.90 (25.30, 31.80) | 29.20 (25.80, 33.35) | 30.00 (27.00, 33.80) | <0.001 |
| Waist circumference (cm) | 93.81 (12.37) | 86.75 (10.93) | 91.98 (10.92) | 96.74 (11.51) | 100.35 (11.72) | <0.001 | 99.06 (14.67) | 91.78 (13.27) | 97.66 (13.97) | 101.61 (14.43) | 105.19 (13.51) | <0.001 |
| Height (m) | 1.66 (0.09) | 1.63 (0.08) | 1.65 (0.09) | 1.68 (0.09) | 1.69 (0.09) | <0.001 | 1.65 (0.10) | 1.63 (0.09) | 1.64 (0.10) | 1.66 (0.10) | 1.68 (0.10) | <0.001 |
| Weight (kg) | 76.21 (14.55) | 68.44 (11.97) | 74.06 (12.96) | 79.73 (13.42) | 83.22 (15.23) | <0.001 | 80.44 (17.38) | 73.26 (14.92) | 78.42 (16.52) | 83.18 (17.86) | 86.91 (17.02) | <0.001 |
| SBP (mmHg) | 132.57 (17.38) | 131.91 (18.32) | 133.07 (17.81) | 131.93 (16.34) | 133.37 (16.91) | 0.436 | 128.65 (19.00) | 126.44 (19.06) | 128.22 (19.00) | 129.84 (19.47) | 130.09 (18.25) | <0.001 |
| DBP (mmHg) | 76.08 (10.36) | 75.93 (10.79) | 76.29 (9.86) | 76.29 (9.90) | 75.81 (10.89) | 0.847 | 79.65 (10.98) | 78.87 (11.36) | 79.58 (10.62) | 80.06 (10.97) | 80.10 (10.93) | 0.086 |
| Dyslipidemia | 1,359 (70.9%) | 329 (66.2%) | 321 (65.6%) | 329 (70.6%) | 380 (81.5%) | <0.001 | 1,935 (60.4%) | 417 (52.1%) | 445 (55.6%) | 430 (53.8%) | 643 (80.3%) | <0.001 |
| Dyslipidemia treatment | NA | NA | NA | NA | NA | NA | 1,128 (35.2%) | 204 (25.5%) | 283 (35.3%) | 318 (39.8%) | 323 (40.3%) | <0.001 |
| Hypertension | 927 (48.3%) | 227 (45.7%) | 225 (46.0%) | 215 (46.1%) | 260 (55.8%) | 0.001 | 1,921 (60.0%) | 406 (50.7%) | 480 (59.9%) | 516 (64.5%) | 519 (64.8%) | <0.001 |
| Hypertension treatment | 213 (11.1%) | 44 (8.9%) | 46 (9.4%) | 47 (10.1%) | 76 (16.3%) | <0.001 | 1,437 (44.9%) | 292 (36.5%) | 350 (43.7%) | 403 (50.4%) | 392 (48.9%) | <0.001 |
| Diabetes | 67 (3.5%) | 0 (0.0%) | 3 (0.6%) | 12 (2.6%) | 52 (11.2%) | <0.001 | 387 (12.1%) | 24 (3.0%) | 53 (6.6%) | 101 (12.6%) | 209 (26.1%) | <0.001 |
| Diabetes treatment | 36 (1.9%) | 0 (0.0%) | 1 (0.2%) | 6 (1.3%) | 29 (6.2%) | <0.001 | 262 (8.2%) | 15 (1.9%) | 33 (4.1%) | 72 (9.0%) | 142 (17.7%) | <0.001 |
| Heart disease | 158 (8.2%) | 33 (6.6%) | 38 (7.8%) | 34 (7.3%) | 53 (11.4%) | 0.037 | 357 (11.1%) | 84 (10.5%) | 86 (10.7%) | 93 (11.6%) | 94 (11.7%) | 0.818 |
| Stroke | 20 (1.0%) | 1 (0.2%) | 4 (0.8%) | 10 (2.1%) | 5 (1.1%) | 0.026 | 36 (1.1%) | 3 (0.4%) | 10 (1.2%) | 14 (1.8%) | 9 (1.1%) | 0.076 |
| Cancer | 91 (4.7%) | 31 (6.2%) | 23 (4.7%) | 15 (3.2%) | 22 (4.7%) | 0.176 | 327 (10.2%) | 68 (8.5%) | 100 (12.5%) | 68 (8.5%) | 91 (11.4%) | 0.011 |
| TC (mg/dL) | 234.03 (44.73) | 252.22 (42.22) | 242.52 (43.85) | 232.08 (39.40) | 207.66 (40.59) | <0.001 | 209.38 (40.05) | 224.26 (38.82) | 214.97 (38.76) | 205.63 (38.43) | 192.66 (37.26) | <0.001 |
| HDL-C (mg/dL) | 60.48 (14.59) | 79.10 (10.58) | 63.24 (4.75) | 54.08 (4.05) | 44.13 (5.56) | <0.001 | 56.92 (16.16) | 78.29 (11.15) | 59.74 (6.38) | 49.81 (5.30) | 39.83 (6.62) | <0.001 |
| HbA1c (%) | 5.44 (0.43) | 5.27 (0.32) | 5.39 (0.31) | 5.44 (0.35) | 5.67 (0.59) | <0.001 | 5.65 (0.69) | 5.36 (0.41) | 5.51 (0.46) | 5.69 (0.55) | 6.04 (0.99) | <0.001 |
| CRP (ug/mL) | 1.70 (0.80, 3.50) | 1.20 (0.60, 2.60) | 1.70 (0.80, 3.10) | 1.80 (0.90, 3.40) | 2.40 (1.10, 4.60) | <0.001 | 1.89 (0.91, 4.27) | 1.51 (0.76, 3.68) | 1.72 (0.87, 3.88) | 2.11 (1.00, 4.78) | 2.32 (1.14, 5.30) | <0.001 |
| LDL-C (mg/dL) | 143.08 (37.72) | 151.05 (37.26) | 151.59 (37.95) | 145.08 (34.17) | 123.65 (34.33) | <0.001 | NA | NA | NA | NA | NA | NA |
| TG (mg/dL) | 132.86 (97.43, 186.00) | 97.43 (70.86, 132.86) | 115.14 (88.57, 177.14) | 141.71 (106.28, 194.85) | 168.28 (124.00, 239.14) | <0.001 | NA | NA | NA | NA | NA | NA |
| FPG (mg/dL) | 89.55 (12.23) | 86.44 (9.25) | 88.47 (10.26) | 90.07 (10.95) | 93.50 (16.36) | <0.001 | NA | NA | NA | NA | NA | NA |
| Cumulative HbA1c/HDL-C ratio | 30.16 (7.77) | 22.08 (3.72) | 27.79 (3.60) | 31.76 (3.90) | 39.69 (6.02) | <0.001 | 33.93 (9.54) | 24.34 (5.17) | 30.49 (4.69) | 35.74 (4.60) | 45.14 (7.83) | <0.001 |
| HbA1c/HDL-C ratio at V1 | 3.69 (0.96) | 2.61 (0.30) | 3.31 (0.18) | 3.89 (0.18) | 5.02 (0.65) | <0.001 | 4.16 (1.30) | 2.69 (0.34) | 3.58 (0.24) | 4.43 (0.26) | 5.94 (0.88) | <0.001 |
| HbA1c/HDL-C ratio at V3 | 3.86 (1.03) | 2.87 (0.58) | 3.59 (0.60) | 4.07 (0.64) | 4.99 (0.90) | <0.001 | 4.25 (1.33) | 3.14 (0.92) | 3.87 (0.90) | 4.47 (0.87) | 5.54 (1.26) | <0.001 |
| HbA1c/HDL-C ratio at V5 | 3.68 (1.16) | 2.69 (0.64) | 3.40 (0.73) | 3.85 (0.84) | 4.84 (1.16) | <0.001 | 4.29 (1.35) | 3.20 (0.96) | 3.92 (0.95) | 4.51 (1.01) | 5.56 (1.21) | <0.001 |
| CMM during follow-up | 303 (15.8%) | 62 (12.5%) | 72 (14.7%) | 69 (14.8%) | 100 (21.5%) | <0.001 | 283 (8.8%) | 44 (5.5%) | 69 (8.6%) | 78 (9.8%) | 92 (11.5%) | <0.001 |

Abbreviations: BMI, body mass index; SBP, systolic blood pressure; DBP, diastolic blood pressure; TC, total cholesterol; HDL-C, high-density lipoprotein cholesterol; CRP, C-reactive protein; LDL-C, low-density lipoprotein cholesterol; TG, triglycerides; FPG, fasting plasma glucose; V1, visit 1; V3, visit 3; V5, visit 5; CMM, cardiometabolic multimorbidity.

Table S12 Patient demographics and baseline characteristics based on trajectory of HbA1c/HDL-C ratio

| **Characteristic** | **ELSA** | | | | **HRS** | | | |
| --- | --- | --- | --- | --- | --- | --- | --- | --- |
|  | **Overall (N = 1,918)** | **Persistently low trajectory (N = 1,783)** | **Persistently high trajectory (N = 135)** | **P-value** | **Overall (N = 3,203)** | **Persistently low trajectory (N = 2,907)** | **Persistently high trajectory (N = 296)** | **P-value** |
| Age | 61.00 (57.00, 68.00) | 61.00 (57.00, 68.00) | 61.00 (56.00, 69.00) | 0.644 | 65.06 (8.13) | 65.10 (8.13) | 64.61 (8.17) | 0.324 |
| Gender |  |  |  | <0.001 |  |  |  | <0.001 |
| Male | 817 (42.6) | 724 (40.6) | 93 (68.9) |  | 1,149 (35.9) | 1,001 (34.4) | 148 (50.0) |  |
| Female | 1,101 (57.4) | 1,059 (59.4) | 42 (31.1) |  | 2,054 (64.1) | 1,906 (65.6) | 148 (50.0) |  |
| Education |  |  |  | 0.041 |  |  |  | 0.002 |
| Below high school | 1,084 (56.5) | 997 (55.9) | 87 (64.4) |  | 434 (13.5) | 381 (13.1) | 53 (17.9) |  |
| High school | 160 (8.3) | 146 (8.2) | 14 (10.4) |  | 1,126 (35.2) | 1,010 (34.7) | 116 (39.2) |  |
| Above high school | 674 (35.1) | 640 (35.9) | 34 (25.2) |  | 1,643 (51.3) | 1,516 (52.1) | 127 (42.9) |  |
| Marital_status |  |  |  | 0.112 |  |  |  | 0.202 |
| Married | 1,390 (72.5) | 1,284 (72.0) | 106 (78.5) |  | 2,255 (70.4) | 2,037 (70.1) | 218 (73.6) |  |
| Others | 528 (27.5) | 499 (28.0) | 29 (21.5) |  | 948 (29.6) | 870 (29.9) | 78 (26.4) |  |
| Residence |  |  |  | NA |  |  |  | 0.417 |
| Urban | NA | NA | NA |  | 2,141 (66.8) | 1,950 (67.1) | 191 (64.5) |  |
| Rural | NA | NA | NA |  | 1,062 (33.2) | 957 (32.9) | 105 (35.5) |  |
| Smoking | 1,143 (59.6) | 1,048 (58.8) | 95 (70.4) | 0.009 | 1,665 (52.0) | 1,506 (51.8) | 159 (53.7) | 0.548 |
| Drinking | 1,775 (92.5) | 1,656 (92.9) | 119 (88.1) | 0.062 | 1,870 (58.4) | 1,717 (59.1) | 153 (51.7) | 0.016 |
| BMI (kg/m^2^) | 27.52 (4.53) | 27.34 (4.47) | 29.84 (4.67) | <0.001 | 28.50 (25.30, 32.30) | 28.30 (25.10, 32.20) | 30.25 (27.30, 33.80) | <0.001 |
| Waist circumference (cm) | 93.81 (12.37) | 93.14 (12.07) | 102.69 (12.96) | <0.001 | 99.06 (14.67) | 98.39 (14.57) | 105.64 (14.00) | <0.001 |
| Height (m) | 1.66 (0.09) | 1.66 (0.09) | 1.69 (0.09) | <0.001 | 1.65 (0.10) | 1.65 (0.10) | 1.68 (0.10) | <0.001 |
| Weight (kg) | 76.21 (14.55) | 75.47 (14.10) | 85.90 (16.84) | <0.001 | 80.44 (17.38) | 79.71 (17.20) | 87.59 (17.53) | <0.001 |
| SBP (mmHg) | 132.57 (17.38) | 132.44 (17.43) | 134.19 (16.66) | 0.260 | 128.65 (19.00) | 128.52 (19.11) | 129.90 (17.84) | 0.232 |
| DBP (mmHg) | 76.08 (10.36) | 76.02 (10.28) | 76.87 (11.42) | 0.361 | 79.65 (10.98) | 79.66 (11.02) | 79.60 (10.64) | 0.937 |
| Dyslipidemia | 1,359 (70.9) | 1,245 (69.8) | 114 (84.4) | 0.001 | 1,935 (60.4) | 1,675 (57.6) | 260 (87.8) | <0.001 |
| Dyslipidemia treatment | NA | NA | NA | NA | 1,128 (35.2) | 1,000 (34.4) | 128 (43.2) | 0.003 |
| Hypertension | 927 (48.3) | 853 (47.8) | 74 (54.8) | 0.126 | 1,921 (60.0) | 1,729 (59.5) | 192 (64.9) | 0.070 |
| Hypertension treatment | 213 (11.1) | 187 (10.5) | 26 (19.3) | 0.003 | 1,437 (44.9) | 1,286 (44.2) | 151 (51.0) | 0.031 |
| Diabetes | 67 (3.5) | 50 (2.8) | 17 (12.6) | <0.001 | 387 (12.1) | 270 (9.3) | 117 (39.5) | <0.001 |
| Diabetes treatment | 36 (1.9) | 24 (1.3) | 12 (8.9) | <0.001 | 262 (8.2) | 183 (6.3) | 79 (26.7) | <0.001 |
| Heart disease | 158 (8.2) | 152 (8.5) | 6 (4.4) | 0.102 | 357 (11.1) | 333 (11.5) | 24 (8.1) | 0.100 |
| Stroke | 20 (1.0) | 20 (1.1) | 0 (0.0) | 0.404 | 36 (1.1) | 35 (1.2) | 1 (0.3) | 0.252 |
| Cancer | 91 (4.7) | 86 (4.8) | 5 (3.7) | 0.671 | 327 (10.2) | 294 (10.1) | 33 (11.1) | 0.635 |
| TC (mg/dL) | 234.03 (44.73) | 234.61 (44.86) | 226.32 (42.43) | 0.038 | 209.38 (40.05) | 211.59 (39.73) | 187.71 (36.58) | <0.001 |
| HDL-C (mg/dL) | 60.48 (14.59) | 61.39 (14.48) | 48.41 (10.03) | <0.001 | 56.92 (16.16) | 58.81 (15.58) | 38.27 (7.68) | <0.001 |
| HbA1c (%) | 5.40 (5.20, 5.60) | 5.40 (5.20, 5.60) | 5.50 (5.30, 5.90) | <0.001 | 5.57 (5.22, 5.91) | 5.47 (5.22, 5.80) | 6.03 (5.59, 6.72) | <0.001 |
| CRP (ug/mL) | 1.70 (0.80, 3.50) | 1.70 (0.80, 3.40) | 2.50 (1.20, 4.60) | <0.001 | 1.89 (0.91, 4.27) | 1.80 (0.89, 4.17) | 2.77 (1.22, 5.66) | <0.001 |
| LDL-C (mg/dL) | 143.08 (37.72) | 143.65 (37.71) | 135.57 (37.16) | 0.016 | NA | NA | NA | NA |
| TG (mg/dL) | 132.86 (97.43, 186.00) | 124.00 (88.57, 177.14) | 186.00 (132.86, 256.85) | <0.001 | NA | NA | NA | NA |
| FPG (mg/dL) | 89.55 (12.23) | 89.38 (12.08) | 91.77 (14.01) | 0.029 | NA | NA | NA | NA |
| Cumulative HbA1c/HDL-C ratio | 30.16 (7.77) | 29.10 (6.82) | 44.19 (5.64) | <0.001 | 33.93 (9.54) | 31.95 (7.38) | 53.31 (5.93) | <0.001 |
| HbA1c/HDL-C ratio at V1 | 3.69 (0.96) | 3.61 (0.91) | 4.71 (0.99) | <0.001 | 4.16 (1.30) | 3.92 (1.06) | 6.54 (1.04) | <0.001 |
| HbA1c/HDL-C ratio at V3 | 3.86 (1.03) | 3.73 (0.93) | 5.57 (0.85) | <0.001 | 4.25 (1.33) | 4.00 (1.08) | 6.72 (1.01) | <0.001 |
| HbA1c/HDL-C ratio at V5 | 3.68 (1.16) | 3.48 (0.93) | 6.25 (0.76) | <0.001 | 4.29 (1.35) | 4.05 (1.13) | 6.68 (1.00) | <0.001 |
| CMM during follow-up | 303 (15.8) | 267 (15.0) | 36 (26.7) | <0.001 | 283 (8.8) | 231 (7.9) | 52 (17.6) | <0.001 |

Abbreviations: BMI, body mass index; SBP, systolic blood pressure; DBP, diastolic blood pressure; TC, total cholesterol; HDL-C, high-density lipoprotein cholesterol; CRP, C-reactive protein; LDL-C, low-density lipoprotein cholesterol; TG, triglycerides; FPG, fasting plasma glucose; V1, visit 1; V3, visit 3; V5, visit 5; CMM, cardiometabolic multimorbidity.

Table S13 Baseline characteristics between inclusion and exclusion participants who provided blood samples

| **Characteristic** | **ELSA** | | | | **HRS** | | | |
| --- | --- | --- | --- | --- | --- | --- | --- | --- |
|  | **Overall (N = 7,666)** | **Excluded (N = 5,748)** | **Included (N = 1,918)** | **P-value** | **Overall (N = 13,064)** | **Excluded (N = 9,861)** | **Included (N = 3,203)** | **P-value** |
| Age | 65.00 (58.00, 74.00) | 67.00 (59.00, 75.00) | 61.00 (57.00, 68.00) | <0.001 | 68.00 (60.00, 75.00) | 69.00 (61.00, 77.00) | 65.00 (58.00, 71.00) | <0.001 |
| Gender |  |  |  | 0.019 |  |  |  | <0.001 |
| Male | 3,451 (45.0%) | 2,634 (45.8%) | 817 (42.6%) |  | 5,287 (40.5%) | 4,138 (42.0%) | 1,149 (35.9%) |  |
| Female | 4,215 (55.0%) | 3,114 (54.2%) | 1,101 (57.4%) |  | 7,777 (59.5%) | 5,723 (58.0%) | 2,054 (64.1%) |  |
| Education |  |  |  | <0.001 |  |  |  | <0.001 |
| Below high school | 4,596 (65.8%) | 3,602 (68.9%) | 994 (56.7%) |  | 2,659 (20.4%) | 2,225 (22.6%) | 434 (13.5%) |  |
| High school | 514 (7.4%) | 364 (7.0%) | 150 (8.6%) |  | 4,699 (36.0%) | 3,573 (36.2%) | 1,126 (35.2%) |  |
| Above high school | 1,871 (26.8%) | 1,262 (24.1%) | 609 (34.7%) |  | 5,704 (43.7%) | 4,061 (41.2%) | 1,643 (51.3%) |  |
| Marital status |  |  |  | <0.001 |  |  |  | <0.001 |
| Married | 5,163 (67.4%) | 3,773 (65.7%) | 1,390 (72.5%) |  | 8,464 (64.8%) | 6,210 (63.0%) | 2,254 (70.4%) |  |
| Others | 2,502 (32.6%) | 1,974 (34.3%) | 528 (27.5%) |  | 4,599 (35.2%) | 3,651 (37.0%) | 948 (29.6%) |  |
| Residence |  |  |  | NA |  |  |  | 0.373 |
| Urban | NA | NA | NA |  | 8,802 (67.5%) | 6,663 (67.7%) | 2,139 (66.8%) |  |
| Rural | NA | NA | NA |  | 4,241 (32.5%) | 3,180 (32.3%) | 1,061 (33.2%) |  |
| Smoking | 4,862 (63.4%) | 3,719 (64.7%) | 1,143 (59.6%) | <0.001 | 7,375 (56.9%) | 5,722 (58.5%) | 1,653 (51.9%) | <0.001 |
| Drinking | 6,134 (89.5%) | 4,449 (88.2%) | 1,685 (93.0%) | <0.001 | 6,722 (51.5%) | 4,852 (49.2%) | 1,870 (58.4%) | <0.001 |
| BMI (kg/m^2^) | 27.93 (4.89) | 28.07 (5.02) | 27.52 (4.48) | <0.001 | 28.50 (25.00, 32.50) | 28.40 (25.00, 32.60) | 28.60 (25.30, 32.30) | 0.153 |
| Waist circumference (cm) | 95.67 (13.17) | 96.32 (13.38) | 93.78 (12.35) | <0.001 | 100.87 (15.46) | 101.46 (15.69) | 99.08 (14.61) | <0.001 |
| Height (m) | 1.65 (0.10) | 1.65 (0.10) | 1.66 (0.09) | <0.001 | 1.65 (0.10) | 1.66 (0.10) | 1.65 (0.10) | 0.522 |
| Weight (kg) | 76.47 (15.66) | 76.56 (16.06) | 76.18 (14.41) | 0.359 | 80.14 (18.01) | 80.08 (18.34) | 80.30 (17.00) | 0.568 |
| SBP (mmHg) | 135.38 (18.99) | 136.34 (19.41) | 132.53 (17.37) | <0.001 | 131.04 (20.57) | 131.84 (21.00) | 128.63 (19.01) | <0.001 |
| DBP (mmHg) | 75.02 (11.27) | 74.67 (11.54) | 76.06 (10.34) | <0.001 | 79.17 (11.61) | 79.03 (11.82) | 79.59 (10.97) | 0.019 |
| Dyslipidemia | 4,492 (71.9%) | 3,133 (72.3%) | 1,359 (70.9%) | 0.239 | 8,048 (68.7%) | 6,113 (71.8%) | 1,935 (60.4%) | <0.001 |
| Dyslipidemia treatment | NA | NA | NA | NA | 5,429 (41.7%) | 4,301 (43.8%) | 1,128 (35.2%) | <0.001 |
| Hypertension | 4,515 (59.5%) | 3,591 (63.2%) | 924 (48.4%) | <0.001 | 8,845 (68.4%) | 6,932 (71.0%) | 1,913 (60.3%) | <0.001 |
| Hypertension treatment | 1,124 (14.7%) | 911 (15.9%) | 213 (11.1%) | <0.001 | 6,834 (52.5%) | 5,400 (55.0%) | 1,434 (44.8%) | <0.001 |
| Diabetes | 747 (9.7%) | 680 (11.8%) | 67 (3.5%) | <0.001 | 3,067 (23.9%) | 2,680 (27.8%) | 387 (12.1%) | <0.001 |
| Diabetes treatment | 500 (6.5%) | 464 (8.1%) | 36 (1.9%) | <0.001 | 2,277 (17.4%) | 2,018 (20.5%) | 259 (8.1%) | <0.001 |
| Heart disease | 1,371 (17.9%) | 1,213 (21.1%) | 158 (8.2%) | <0.001 | 3,174 (24.3%) | 2,817 (28.6%) | 357 (11.1%) | <0.001 |
| Stroke | 341 (4.4%) | 321 (5.6%) | 20 (1.0%) | <0.001 | 986 (7.5%) | 950 (9.6%) | 36 (1.1%) | <0.001 |
| Cancer | 562 (7.3%) | 471 (8.2%) | 91 (4.7%) | <0.001 | 1,853 (14.2%) | 1,526 (15.5%) | 327 (10.2%) | <0.001 |
| TC (mg/dL) | 228.59 (46.58) | 225.97 (47.22) | 234.03 (44.73) | <0.001 | 201.35 (42.06) | 198.49 (42.38) | 209.39 (40.05) | <0.001 |
| HDL (mg/dL) | 58.94 (15.02) | 58.19 (15.17) | 60.48 (14.59) | <0.001 | 54.70 (16.19) | 53.75 (16.11) | 56.92 (16.16) | <0.001 |
| HbA1c (%) | 5.60 (0.73) | 5.68 (0.83) | 5.44 (0.43) | <0.001 | 5.88 (1.00) | 5.95 (1.07) | 5.65 (0.69) | <0.001 |
| CRP (ug/mL) | 2.00 (0.90, 4.20) | 2.20 (1.00, 4.50) | 1.70 (0.80, 3.50) | <0.001 | 2.13 (0.99, 4.90) | 2.22 (1.02, 5.06) | 1.89 (0.91, 4.27) | <0.001 |
| LDL (mg/dL) | 138.68 (38.64) | 136.27 (39.00) | 143.69 (37.40) | <0.001 | NA | NA | NA | NA |
| TG (mg/dL) | 132.86 (97.43, 194.85) | 132.86 (97.43, 194.85) | 132.86 (97.43, 186.00) | <0.001 | NA | NA | NA | NA |
| FPG (mg/dL) | 90.26 (16.78) | 91.15 (19.22) | 88.81 (11.59) | <0.001 | NA | NA | NA | NA |
| Cumulative HbA1c/HDL-C ratio | 24.29 (12.51) | 18.81 (13.57) | 30.16 (7.77) | <0.001 | 36.41 (13.11) | 38.11 (14.83) | 33.93 (9.54) | <0.001 |
| HbA1c/HDL-C ratio at V1 | 3.14 (1.85) | 2.96 (2.03) | 3.69 (0.96) | <0.001 | 4.55 (1.74) | 4.72 (1.87) | 4.16 (1.30) | <0.001 |
| HbA1c/HDL-C ratio at V3 | 3.22 (1.87) | 2.85 (2.14) | 3.86 (1.03) | <0.001 | 4.62 (1.78) | 4.79 (1.93) | 4.25 (1.33) | <0.001 |
| HbA1c/HDL-C ratio at V5 | 2.40 (2.85) | 1.38 (3.34) | 3.68 (1.16) | <0.001 | 4.58 (1.71) | 4.73 (1.86) | 4.29 (1.35) | <0.001 |
| CMM during follow-up | 820 (10.7%) | 517 (9.0%) | 303 (15.8%) | <0.001 | 1,701 (13.0%) | 1,418 (14.4%) | 283 (8.8%) | <0.001 |

Abbreviations: BMI, body mass index; SBP, systolic blood pressure; DBP, diastolic blood pressure; TC, total cholesterol; HDL-C, high-density lipoprotein cholesterol; CRP, C-reactive protein; LDL-C, low-density lipoprotein cholesterol; TG, triglycerides; FPG, fasting plasma glucose; V1, visit 1; V3, visit 3; V5, visit 5; CMM, cardiometabolic multimorbidity.

Table S14 Predictive performance of HbA1c/HDL-C ratio for CMM.

| Cohort | Model | AUC (95%CI) | NRI (95% CI) | IDI (95% CI) |
| --- | --- | --- | --- | --- |
| ELSA | Basic | 0.598 (0.563-0.633) | Ref | Ref |
|  | Basic + Cumulative HbA1c/HDL-C ratio | 0.611 (0.576-0.646) | 0.103(-0.022,0.239) | 0.006(0.001,0.015)* |
|  | Basic + HbA1c/HDL-C ratio at V1 | 0.601 (0.566-0.635) | -0.004(-0.117,0.139) | 0.002(-0.000,0.007) |
|  | Basic + Trajectory of HbA1c/HDL-C ratio | 0.605 (0.570-0.641) | 0.028(-0.093,0.170) | 0.003(-0.000,0.013) |
| HRS | Basic | 0.655 (0.624-0.687) | Ref | Ref |
|  | Basic + Cumulative HbA1c/HDL-C ratio | 0.678 (0.648-0.709)* | 0.269(0.111,0.370)* | 0.012(0.004,0.027)* |
|  | Basic + HbA1c/HDL-C ratio at V1 | 0.670 (0.640-0.701) | 0.192(0.068,0.319)* | 0.009(0.002,0.022)* |
|  | Basic + Trajectory of HbA1c/HDL-C ratio | 0.680 (0.649-0.712)* | 0.181(0.016,0.307)* | 0.010(0.003,0.025)* |

Note: Basic model included age, sex, marital status, educational level, smoking status, drinking status, WC, dyslipidemia, hypertension, cancer, TC, and CRP. * indicate p<0.05.

Abbreviations: CMM, cardiometabolic multimorbidity; AUC, area under the curve; NRI, net reclassification improvement; IDI, integrated discrimination improvement; V1, visit 1.

Table S15 Mediation analysis of BMI in the association between HbA1c/HDL-C ratio and newly-onset CMM

| Cohort | Exposure | Total Effect (95% CI) | P-value | Direct Effect (95% CI) | P-value | Indirect Effect (95% CI) | P-value | Proportion Mediated (95% CI) | P-value |
| --- | --- | --- | --- | --- | --- | --- | --- | --- | --- |
| ELSA | Cumulative HbA1c/HDL-C ratio | -13.2822 (-35.4802, -2.7987) | <0.001 | -13.3031 (-34.9496, -2.5826) | <0.001 | 0.0242 (-2.2004, 2.1783) | 0.984 | -0.27 (-20.17, 18.71) | 0.984 |
|  | HbA1c/HDL-C ratio at V1 | -43.4533 (-127.7355, -1.7420) | 0.028 | -41.5526 (-123.6512, -0.0486) | 0.050 | -2.1943 (-14.0216, 9.2610) | 0.652 | 6.44 (-35.87, 59.13) | 0.664 |
|  | Trajectory of HbA1c/HDL-C ratio | -55.8434 (-94.3428, -21.9563) | 0.002 | -54.4723 (-93.7171, -19.0592) | 0.002 | -2.4280 (-10.9686, 5.3565) | 0.528 | 4.39 (-9.82, 23.99) | 0.530 |
| HRS | Cumulative HbA1c/HDL-C ratio | -9.7899 (-21.4276, -3.4098) | <0.001 | -9.3144 (-20.6884, -3.1395) | <0.001 | -0.4917 (-1.3535, 0.0908) | 0.114 | 5.1 (-1.11, 12.6) | 0.114 |
|  | HbA1c/HDL-C ratio at V1 | -43.2142 (-88.7722, -13.9716) | <0.001 | -40.7074 (-83.2177, -12.7265) | <0.001 | -3.0850 (-8.0957, 0.3247) | 0.104 | 7.32 (-1.26, 18.35) | 0.104 |
|  | Trajectory of HbA1c/HDL-C ratio | -50.9182 (-80.1391, -30.0823) | <0.001 | -50.0776 (-79.2955, -29.2679) | <0.001 | -1.8463 (-4.4322, -0.1363) | 0.034 | 3.33 (0.28, 8.31) | 0.034 |

Note: Adjusted for age, sex, marital status, educational level, smoking status, drinking status, dyslipidemia, hypertension, cancer, TC, and CRP.

Abbreviations: CMM, cardiometabolic multimorbidity; V1, visit 1.

Table S16 Multivariate logistic regression of the relationships between HbA1c/HDL-C ratio and the risk of incident CMM in ELSA and HRS

| **Characteristic** | **ELSA** | | | | | | **HRS** | | | | | |
| --- | --- | --- | --- | --- | --- | --- | --- | --- | --- | --- | --- | --- |
|  | **Model 1** | | **Model 2** | | **Model 3** | | **Model 1** | | **Model 2** | | **Model 3** | |
|  | **OR (95% CI)** | **P-value** | **OR (95% CI)** | **P-value** | **OR (95% CI)** | **P-value** | **OR (95% CI)** | **P-value** | **OR (95% CI)** | **P-value** | **OR (95% CI)** | **P-value** |
| **Cumulative HbA1c/HDL-C ratio** | | | | | | | | | | | | |
| **Per 1 SD** | 1.35 (1.19, 1.54) | <0.001 | 1.33 (1.16, 1.52) | <0.001 | 1.28 (1.11, 1.48) | <0.001 | 1.44 (1.29, 1.62) | <0.001 | 1.37 (1.21, 1.54) | <0.001 | 1.42 (1.24, 1.62) | <0.001 |
| **Quartile** |  |  |  |  |  |  |  |  |  |  |  |  |
| **Q1** | Ref | | Ref | | Ref | | Ref | | Ref | | Ref | |
| **Q2** | 0.73 (0.49, 1.08) | 0.118 | 0.74 (0.49, 1.09) | 0.128 | 0.72 (0.48, 1.07) | 0.107 | 1.02 (0.68, 1.53) | 0.921 | 0.92 (0.61, 1.39) | 0.701 | 0.90 (0.60, 1.36) | 0.624 |
| **Q3** | 1.33 (0.93, 1.91) | 0.115 | 1.29 (0.89, 1.88) | 0.179 | 1.23 (0.84, 1.79) | 0.286 | 1.69 (1.17, 2.46) | 0.005 | 1.44 (0.99, 2.12) | 0.062 | 1.40 (0.95, 2.07) | 0.091 |
| **Q4** | 1.67 (1.17, 2.39) | 0.005 | 1.56 (1.06, 2.30) | 0.023 | 1.42 (0.95, 2.13) | 0.093 | 2.11 (1.47, 3.06) | <0.001 | 1.72 (1.18, 2.54) | 0.006 | 1.76 (1.18, 2.63) | 0.006 |
| **P for trend** |  | <0.001 |  | 0.003 |  | 0.019 |  | <0.001 |  | <0.001 |  | <0.001 |
| **HbA1c/HDL-C ratio at V1** | | | | | | | | | | | | |
| **Per 1 SD** | 1.27 (1.12, 1.43) | <0.001 | 1.23 (1.08, 1.41) | 0.002 | 1.17 (1.00, 1.36) | 0.048 | 1.37 (1.22, 1.54) | <0.001 | 1.28 (1.13, 1.45) | <0.001 | 1.34 (1.17, 1.53) | <0.001 |
| **Quartile** |  |  |  |  |  |  |  |  |  |  |  |  |
| **Q1** | Ref | | Ref | | Ref | | Ref | | Ref | | Ref | |
| **Q2** | 1.21 (0.84, 1.75) | 0.308 | 1.18 (0.82, 1.72) | 0.372 | 1.16 (0.80, 1.69) | 0.426 | 1.58 (1.07, 2.36) | 0.022 | 1.42 (0.96, 2.13) | 0.085 | 1.40 (0.94, 2.10) | 0.101 |
| **Q3** | 1.23 (0.84, 1.80) | 0.283 | 1.17 (0.79, 1.73) | 0.439 | 1.11 (0.75, 1.66) | 0.603 | 1.82 (1.24, 2.70) | 0.002 | 1.54 (1.04, 2.30) | 0.035 | 1.48 (0.99, 2.24) | 0.056 |
| **Q4** | 1.92 (1.34, 2.78) | <0.001 | 1.77 (1.21, 2.62) | 0.004 | 1.56 (1.02, 2.40) | 0.041 | 2.20 (1.51, 3.25) | <0.001 | 1.77 (1.19, 2.67) | 0.005 | 1.84 (1.21, 2.82) | 0.005 |
| **P for trend** |  | <0.001 |  | 0.005 |  | 0.064 |  | <0.001 |  | 0.007 |  | 0.006 |
| **Trajectory of HbA1c/HDL-C ratio** | | | | | | | | | | | | |
| **Persistently low trajectory** | Ref | | Ref | | Ref | | Ref | | Ref | | Ref | |
| **Persistently high trajectory** | 2.01 (1.32, 3.01) | <0.001 | 1.86 (1.21, 2.81) | 0.004 | 1.82 (1.18, 2.75) | 0.006 | 2.46 (1.75, 3.40) | <0.001 | 2.21 (1.57, 3.07) | <0.001 | 2.44 (1.69, 3.47) | <0.001 |

Note: Model 1 included adjustment for age and sex. Model 2 was further adjusted for marital status, educational level, smoking status, drinking status and WC. Model 3 additionally controlled for dyslipidemia, hypertension, cancer, TC, and CRP.

Abbreviations: CMM, cardiometabolic multimorbidity; V1, visit 1.

Table S17 Pooled analysis of associations between HbA1c/HDL-C ratio and CMM risk in different logistic regression models

| **Pooled analysis** | | | | | | | | | | | | |
| --- | --- | --- | --- | --- | --- | --- | --- | --- | --- | --- | --- | --- |
| **Characteristic** | **Model 1** | | | | **Model 2** | | | | **Model 3** | | | |
|  | **OR (95% CI)** | **P-value*** | **I^2^ (%)** | **P-value^#^** | **OR (95% CI)** | **P-value*** | **I^2^ (%)** | **P-value^#^** | **OR (95% CI)** | **P-value*** | **I^2^ (%)** | **P-value^#^** |
| **Cumulative HbA1c/HDL-C ratio** | | | | | | | | | | | | |
| **Per 1 SD** | 1.40 (1.29, 1.53) | <0.001 | 0.0 | 0.464 | 1.35 (1.23, 1.48) | <0.001 | 0.0 | 0.746 | 1.35 (1.23, 1.49) | <0.001 | 0.0 | 0.323 |
| **Quartile** |  |  |  |  |  |  |  |  |  |  |  |  |
| **Q1** | Ref | | | | Ref | | | | Ref | | | |
| **Q2** | 0.86 (0.62, 1.19) | 0.367 | 25.5 | 0.247 | 0.82 (0.62, 1.09) | 0.174 | 0.0 | 0.433 | 0.80 (0.60, 1.07) | 0.134 | 0.0 | 0.443 |
| **Q3** | 1.50 (1.16, 1.94) | 0.002 | 0.0 | 0.366 | 1.36 (1.04, 1.78) | 0.024 | 0.0 | 0.690 | 1.31 (1.00, 1.71) | 0.052 | 0.0 | 0.639 |
| **Q4** | 1.87 (1.45, 2.41) | <0.001 | 0.0 | 0.364 | 1.64 (1.25, 2.15) | <0.001 | 0.0 | 0.724 | 1.58 (1.19, 2.10) | 0.002 | 0.0 | 0.462 |
| **P for trend** |  | <0.001 |  |  |  | <0.001 |  |  |  | <0.001 |  |  |
| **HbA1c/HDL-C ratio at V1** | | | | | | | | | | | | |
| **Per 1 SD** | 1.32 (1.21, 1.44) | <0.001 | 0.0 | 0.376 | 1.26 (1.15, 1.38) | <0.001 | 0.0 | 0.658 | 1.26 (1.10, 1.44) | <0.001 | 42.6 | 0.187 |
| **Quartile** |  |  |  |  |  |  |  |  |  |  |  |  |
| **Q1** | Ref | | | | Ref | | | | Ref | | | |
| **Q2** | 1.37 (1.05, 1.79) | 0.021 | 0.0 | 0.326 | 1.29 (0.98, 1.69) | 0.067 | 0.0 | 0.516 | 1.27 (0.96, 1.67) | 0.089 | 0.0 | 0.512 |
| **Q3** | 1.49 (1.02, 2.19) | 0.041 | 50.5 | 0.155 | 1.33 (1.01, 1.76) | 0.043 | 0.0 | 0.334 | 1.28 (0.96, 1.70) | 0.087 | 0.0 | 0.317 |
| **Q4** | 2.05 (1.57, 2.67) | <0.001 | 0.0 | 0.617 | 1.77 (1.34, 2.34) | <0.001 | 0.0 | 0.996 | 1.70 (1.26, 2.29) | <0.001 | 0.0 | 0.598 |
| **P for trend** |  | <0.001 |  |  |  | <0.001 |  |  |  | 0.001 |  |  |
| **Trajectory of HbA1c/HDL-C ratio** | | | | | | | | | | | | |
| **Persistently low trajectory** | Ref | | | | Ref | | | | Ref | | | |
| **Persistently high trajectory** | 2.27 (1.76, 2.94) | <0.001 | 0.0 | 0.456 | 2.07 (1.59, 2.69) | <0.001 | 0.0 | 0.531 | 2.15 (1.62, 2.87) | <0.001 | 8.2 | 0.297 |

Note: Model 1 included adjustment for age and sex. Model 2 was further adjusted for marital status, educational level, smoking status, drinking status and WC. Model 3 additionally controlled for dyslipidemia, hypertension, cancer, TC, and CRP. P-value* for the pooled HR 95%CI. P-value^#^ for the heterogeneity.

Abbreviations: CMM, cardiometabolic multimorbidity; V1, visit 1.

Table S18 Multivariate Cox regression of the relationships between HbA1c/HDL-C ratio and the risk of incident CMM in ELSA and HRS after excluding missing covariates

| **Characteristic** | **ELSA** | | | | | | **HRS** | | | | | |
| --- | --- | --- | --- | --- | --- | --- | --- | --- | --- | --- | --- | --- |
|  | **Model 1** | | **Model 2** | | **Model 3** | | **Model 1** | | **Model 2** | | **Model 3** | |
|  | **HR (95% CI)** | **P-value** | **HR (95% CI)** | **P-value** | **HR (95% CI)** | **P-value** | **HR (95% CI)** | **P-value** | **HR (95% CI)** | **P-value** | **HR (95% CI)** | **P-value** |
| **Cumulative HbA1c/HDL-C ratio** | | | | | | | | | | | | |
| **Per 1 SD** | 1.31 (1.16, 1.49) | <0.001 | 1.29 (1.13, 1.48) | <0.001 | 1.23 (1.07, 1.42) | 0.004 | 1.45 (1.30, 1.62) | <0.001 | 1.37 (1.22, 1.54) | <0.001 | 1.40 (1.23, 1.59) | <0.001 |
| **Quartile** |  |  |  |  |  |  |  |  |  |  |  |  |
| **Q1** | Ref | | Ref | | Ref | | Ref | | Ref | | Ref | |
| **Q2** | 0.77 (0.52, 1.14) | 0.193 | 0.78 (0.52, 1.16) | 0.222 | 0.76 (0.51, 1.14) | 0.184 | 1.03 (0.69, 1.54) | 0.879 | 0.93 (0.62, 1.40) | 0.722 | 0.91 (0.60, 1.37) | 0.649 |
| **Q3** | 1.32 (0.92, 1.88) | 0.134 | 1.29 (0.89, 1.87) | 0.175 | 1.22 (0.84, 1.78) | 0.292 | 1.73 (1.20, 2.49) | 0.004 | 1.46 (1.00, 2.14) | 0.048 | 1.41 (0.96, 2.06) | 0.080 |
| **Q4** | 1.53 (1.07, 2.19) | 0.021 | 1.45 (0.98, 2.13) | 0.060 | 1.28 (0.86, 1.92) | 0.226 | 2.09 (1.46, 3.01) | <0.001 | 1.68 (1.15, 2.46) | 0.008 | 1.67 (1.12, 2.48) | 0.011 |
| **P for trend** |  | 0.002 |  | 0.011 |  | 0.068 |  | <0.001 |  | <0.001 |  | 0.001 |
| **HbA1c/HDL-C ratio at V1** | | | | | | | | | | | | |
| **Per 1 SD** | 1.28 (1.13, 1.45) | <0.001 | 1.26 (1.10, 1.43) | <0.001 | 1.19 (1.03, 1.39) | 0.020 | 1.37 (1.22, 1.53) | <0.001 | 1.28 (1.14, 1.45) | <0.001 | 1.33 (1.17, 1.51) | <0.001 |
| **Quartile** |  |  |  |  |  |  |  |  |  |  |  |  |
| **Q1** | Ref | | Ref | | Ref | | Ref | | Ref | | Ref | |
| **Q2** | 1.32 (0.91, 1.91) | 0.148 | 1.31 (0.90, 1.91) | 0.153 | 1.30 (0.90, 1.90) | 0.167 | 1.64 (1.10, 2.43) | 0.014 | 1.45 (0.97, 2.17) | 0.067 | 1.43 (0.96, 2.13) | 0.079 |
| **Q3** | 1.14 (0.77, 1.69) | 0.506 | 1.11 (0.74, 1.66) | 0.606 | 1.06 (0.71, 1.60) | 0.769 | 1.91 (1.30, 2.82) | 0.001 | 1.61 (1.08, 2.39) | 0.019 | 1.54 (1.03, 2.30) | 0.035 |
| **Q4** | 1.92 (1.33, 2.78) | <0.001 | 1.82 (1.23, 2.68) | 0.003 | 1.59 (1.04, 2.42) | 0.033 | 2.24 (1.52, 3.30) | <0.001 | 1.79 (1.19, 2.68) | 0.005 | 1.82 (1.19, 2.77) | 0.005 |
| **P for trend** |  | 0.001 |  | 0.007 |  | 0.082 |  | <0.001 |  | 0.006 |  | 0.007 |
| **Trajectory of HbA1c/HDL-C ratio** | | | | | | | | | | | | |
| **Persistently low trajectory** | Ref | | Ref | | Ref | | Ref | | Ref | | Ref | |
| **Persistently high trajectory** | 1.73 (1.15, 2.59) | 0.008 | 1.58 (1.04, 2.39) | 0.032 | 1.52 (1.00, 2.31) | 0.050 | 2.49 (1.80, 3.46) | <0.001 | 2.19 (1.57, 3.05) | <0.001 | 2.37 (1.67, 3.36) | <0.001 |

Note: Model 1 included adjustment for age and sex. Model 2 was further adjusted for marital status, educational level, smoking status, drinking status and WC. Model 3 additionally controlled for dyslipidemia, hypertension, cancer, TC, and CRP.

Abbreviations: CMM, cardiometabolic multimorbidity; V1, visit 1.

Table S19 Pooled analysis of associations between HbA1c/HDL-C ratio and CMM risk in different models after excluding missing covariates

| **Pooled analysis** | | | | | | | | | | | | |
| --- | --- | --- | --- | --- | --- | --- | --- | --- | --- | --- | --- | --- |
| **Characteristic** | **Model 1** | | | | **Model 2** | | | | **Model 3** | | | |
|  | **HR (95% CI)** | **P-value*** | **I^2^ (%)** | **P-value^#^** | **HR (95% CI)** | **P-value*** | **I^2^ (%)** | **P-value^#^** | **HR (95% CI)** | **P-value*** | **I^2^ (%)** | **P-value^#^** |
| **Cumulative HbA1c/HDL-C ratio** | | | | | | | | | | | | |
| **Per 1 SD** | 1.39 (1.25, 1.53) | <0.001 | 31.6 | 0.227 | 1.34 (1.22, 1.46) | <0.001 | 0.0 | 0.515 | 1.32 (1.17, 1.49) | <0.001 | 41.5 | 0.191 |
| **Quartile** |  |  |  |  |  |  |  |  |  |  |  |  |
| **Q1** | Ref | | | | Ref | | | | Ref | | | |
| **Q2** | 0.89 (0.67, 1.19) | 0.421 | 3.9 | 0.308 | 0.85 (0.64, 1.13) | 0.263 | 0.0 | 0.547 | 0.83 (0.62, 1.11) | 0.206 | 0.0 | 0.545 |
| **Q3** | 1.50 (1.15, 1.96) | 0.003 | 7.0 | 0.300 | 1.37 (1.05, 1.79) | 0.019 | 0.0 | 0.641 | 1.31 (1.00, 1.71) | 0.048 | 0.0 | 0.607 |
| **Q4** | 1.79 (1.31, 2.44) | <0.001 | 32.1 | 0.225 | 1.56 (1.19, 2.05) | 0.001 | 0.0 | 0.597 | 1.47 (1.11, 1.95) | 0.008 | 0.0 | 0.363 |
| **P for trend** |  | <0.001 |  |  |  | <0.001 |  |  |  | <0.001 |  |  |
| **HbA1c/HDL-C ratio at V1** | | | | | | | | | | | | |
| **Per 1 SD** | 1.33 (1.22, 1.44) | <0.001 | 0.0 | 0.416 | 1.27 (1.16, 1.39) | <0.001 | 0.0 | 0.825 | 1.27 (1.14, 1.40) | <0.001 | 10.3 | 0.291 |
| **Quartile** |  |  |  |  |  |  |  |  |  |  |  |  |
| **Q1** | Ref | | | | Ref | | | | Ref | | | |
| **Q2** | 1.46 (1.11, 1.91) | 0.006 | 0.0 | 0.428 | 1.38 (1.05, 1.81) | 0.022 | 0.0 | 0.720 | 1.36 (1.04, 1.79) | 0.027 | 0.0 | 0.739 |
| **Q3** | 1.48 (0.89, 2.45) | 0.129 | 70.1 | 0.067 | 1.34 (0.93, 1.92) | 0.114 | 38.8 | 0.201 | 1.28 (0.89, 1.84) | 0.181 | 37.9 | 0.204 |
| **Q4** | 2.07 (1.58, 2.70) | <0.001 | 0.0 | 0.570 | 1.80 (1.36, 2.39) | <0.001 | 0.0 | 0.953 | 1.70 (1.26, 2.29) | <0.001 | 0.0 | 0.656 |
| **P for trend** |  | <0.001 |  |  |  | <0.001 |  |  |  | 0.002 |  |  |
| **Trajectory of HbA1c/HDL-C ratio** | | | | | | | | | | | | |
| **Persistently low trajectory** | Ref | | | | Ref | | | | Ref | | | |
| **Persistently high trajectory** | 2.12 (1.48, 3.03) | <0.001 | 47.9 | 0.166 | 1.90 (1.39, 2.61) | <0.001 | 30.7 | 0.230 | 1.93 (1.25, 2.98) | 0.003 | 61.2 | 0.108 |

Note: Model 1 included adjustment for age and sex. Model 2 was further adjusted for marital status, educational level, smoking status, drinking status and WC. Model 3 additionally controlled for dyslipidemia, hypertension, cancer, TC, and CRP. P-value* for the pooled HR 95%CI. P-value^#^ for the heterogeneity.

Abbreviations: CMM, cardiometabolic multimorbidity; V1, visit 1.

Table S20 Multivariate Cox regression of the relationships between HbA1c/HDL-C ratio and the risk of incident CMM in ELSA and HRS after excluding subjects receiving medications for heart disease, stroke, diabetes, hypertension, or dyslipidemia at baseline

| **Characteristic** | **ELSA** | | | | | | **HRS** | | | | | |
| --- | --- | --- | --- | --- | --- | --- | --- | --- | --- | --- | --- | --- |
|  | **Model 1** | | **Model 2** | | **Model 3** | | **Model 1** | | **Model 2** | | **Model 3** | |
|  | **HR (95% CI)** | **P-value** | **HR (95% CI)** | **P-value** | **HR (95% CI)** | **P-value** | **HR (95% CI)** | **P-value** | **HR (95% CI)** | **P-value** | **HR (95% CI)** | **P-value** |
| **Cumulative HbA1c/HDL-C ratio** | | | | | | | | | | | | |
| **Per 1 SD** | 1.62 (1.33, 1.98) | <0.001 | 1.56 (1.27, 1.92) | <0.001 | 1.45 (1.16, 1.81) | <0.001 | 1.36 (0.86, 2.14) | 0.186 | 1.46 (0.90, 2.35) | 0.122 | 1.58 (0.96, 2.60) | 0.070 |
| **Quartile** |  |  |  |  |  |  |  |  |  |  |  |  |
| **Q1** | Ref | | Ref | | Ref | | Ref | | Ref | | Ref | |
| **Q2** | 1.10 (0.63, 1.92) | 0.733 | 1.07 (0.61, 1.89) | 0.802 | 1.06 (0.60, 1.86) | 0.842 | 0.39 (0.08, 1.96) | 0.254 | 0.41 (0.08, 2.07) | 0.279 | 0.36 (0.07, 1.89) | 0.226 |
| **Q3** | 1.76 (1.03, 2.99) | 0.037 | 1.61 (0.93, 2.79) | 0.089 | 1.51 (0.87, 2.63) | 0.147 | 1.95 (0.64, 5.97) | 0.240 | 2.17 (0.66, 7.12) | 0.203 | 2.27 (0.68, 7.60) | 0.184 |
| **Q4** | 2.84 (1.66, 4.87) | <0.001 | 2.61 (1.49, 4.56) | <0.001 | 2.18 (1.21, 3.94) | 0.010 | 1.42 (0.38, 5.33) | 0.604 | 1.85 (0.45, 7.53) | 0.392 | 2.26 (0.52, 9.86) | 0.278 |
| **P for trend** |  | <0.001 |  | <0.001 |  | 0.007 |  | 0.259 |  | 0.161 |  | 0.086 |
| **HbA1c/HDL-C ratio at V1** | | | | | | | | | | | | |
| **Per 1 SD** | 1.53 (1.25, 1.86) | <0.001 | 1.47 (1.20, 1.81) | <0.001 | 1.35 (1.07, 1.72) | 0.012 | 1.27 (0.79, 2.03) | 0.324 | 1.37 (0.83, 2.28) | 0.223 | 1.68 (0.99, 2.84) | 0.054 |
| **Quartile** |  |  |  |  |  |  |  |  |  |  |  |  |
| **Q1** | Ref | | Ref | | Ref | | Ref | | Ref | | Ref | |
| **Q2** | 1.33 (0.77, 2.31) | 0.302 | 1.24 (0.71, 2.16) | 0.441 | 1.22 (0.70, 2.13) | 0.477 | 3.37 (0.87, 13.10) | 0.080 | 3.85 (0.95, 15.66) | 0.059 | 4.37 (1.07, 17.83) | 0.040 |
| **Q3** | 1.82 (1.06, 3.13) | 0.031 | 1.66 (0.95, 2.91) | 0.075 | 1.52 (0.86, 2.69) | 0.148 | 3.81 (0.93, 15.55) | 0.063 | 4.65 (1.07, 20.21) | 0.040 | 5.38 (1.20, 24.12) | 0.028 |
| **Q4** | 2.69 (1.55, 4.69) | <0.001 | 2.42 (1.37, 4.29) | 0.002 | 1.95 (1.03, 3.68) | 0.039 | 2.29 (0.44, 11.81) | 0.323 | 2.95 (0.52, 16.62) | 0.219 | 5.51 (0.88, 34.45) | 0.068 |
| **P for trend** |  | <0.001 |  | 0.002 |  | 0.032 |  | 0.226 |  | 0.158 |  | 0.034 |
| **Trajectory of HbA1c/HDL-C ratio** | | | | | | | | | | | | |
| **Persistently low trajectory** | Ref | | Ref | | Ref | | Ref | | Ref | | Ref | |
| **Persistently high trajectory** | 2.40 (1.25, 4.62) | 0.009 | 2.19 (1.13, 4.23) | 0.020 | 2.03 (1.05, 3.94) | 0.036 | 2.98 (0.84, 10.50) | 0.090 | 3.12 (0.86, 11.36) | 0.084 | 4.01 (0.99, 16.16) | 0.051 |

Note: Model 1 included adjustment for age and sex. Model 2 was further adjusted for marital status, educational level, smoking status, drinking status and WC. Model 3 additionally controlled for dyslipidemia, hypertension, cancer, TC, and CRP.

Abbreviations: CMM, cardiometabolic multimorbidity; V1, visit 1.

Table S21 Pooled analysis of associations between HbA1c/HDL-C ratio and CMM risk in different models after excluding subjects receiving medications for heart disease, stroke, diabetes, hypertension, or dyslipidemia at baseline

| **Pooled analysis** | | | | | | | | | | | | |
| --- | --- | --- | --- | --- | --- | --- | --- | --- | --- | --- | --- | --- |
| **Characteristic** | **Model 1** | | | | **Model 2** | | | | **Model 3** | | | |
|  | **HR (95% CI)** | **P-value*** | **I^2^ (%)** | **P-value^#^** | **HR (95% CI)** | **P-value*** | **I^2^ (%)** | **P-value^#^** | **HR (95% CI)** | **P-value*** | **I^2^ (%)** | **P-value^#^** |
| **Cumulative HbA1c/HDL-C ratio** | | | | | | | | | | | | |
| **Per 1 SD** | 1.58 (1.32, 1.89) | <0.001 | 0.0 | 0.482 | 1.54 (1.28, 1.87) | <0.001 | 0.0 | 0.800 | 1.47 (1.20, 1.81) | <0.001 | 0.0 | 0.758 |
| **Quartile** |  |  |  |  |  |  |  |  |  |  |  |  |
| **Q1** | Ref | | | | Ref | | | | Ref | | | |
| **Q2** | 0.88 (0.38, 2.03) | 0.758 | 29.3 | 0.234 | 0.90 (0.44, 1.87) | 0.786 | 18.1 | 0.269 | 0.83 (0.34, 2.02) | 0.675 | 31.7 | 0.226 |
| **Q3** | 1.79 (1.11, 2.90) | 0.017 | 0.0 | 0.868 | 1.70 (1.03, 2.79) | 0.038 | 0.0 | 0.657 | 1.62 (0.98, 2.69) | 0.061 | 0.0 | 0.548 |
| **Q4** | 2.57 (1.56, 4.24) | <0.001 | 0.0 | 0.341 | 2.49 (1.48, 4.18) | <0.001 | 0.0 | 0.656 | 2.19 (1.27, 3.79) | 0.005 | 0.0 | 0.965 |
| **P for trend** |  | <0.001 |  |  |  | <0.001 |  |  |  | 0.002 |  |  |
| **HbA1c/HDL-C ratio at V1** | | | | | | | | | | | | |
| **Per 1 SD** | 1.48 (1.24, 1.78) | <0.001 | 0.0 | 0.471 | 1.46 (1.21, 1.76) | <0.001 | 0.0 | 0.796 | 1.40 (1.13, 1.74) | 0.002 | 0.0 | 0.465 |
| **Quartile** |  |  |  |  |  |  |  |  |  |  |  |  |
| **Q1** | Ref | | | | Ref | | | | Ref | | | |
| **Q2** | 1.71 (0.77, 3.81) | 0.191 | 34.9 | 0.215 | 1.81 (0.64, 5.13) | 0.266 | 53.8 | 0.141 | 1.95 (0.59, 6.47) | 0.276 | 63.2 | 0.099 |
| **Q3** | 2.00 (1.20, 3.33) | 0.007 | 0.0 | 0.337 | 2.20 (0.90, 5.42) | 0.085 | 39.4 | 0.199 | 2.35 (0.73, 7.61) | 0.154 | 58.0 | 0.123 |
| **Q4** | 2.65 (1.57, 4.48) | <0.001 | 0.0 | 0.854 | 2.47 (1.43, 4.25) | 0.001 | 0.0 | 0.831 | 2.26 (1.11, 4.59) | 0.024 | 8.9 | 0.295 |
| **P for trend** |  | <0.001 |  |  |  | <0.001 |  |  |  | 0.021 |  |  |
| **Trajectory of HbA1c/HDL-C ratio** | | | | | | | | | | | | |
| **Persistently low trajectory** | Ref | | | | Ref | | | | Ref | | | |
| **Persistently high trajectory** | 2.51 (1.40, 4.49) | 0.002 | 0.0 | 0.766 | 2.36 (1.31, 4.24) | 0.004 | 0.0 | 0.631 | 2.30 (1.27, 4.19) | 0.006 | 0.0 | 0.389 |

Note: Model 1 included adjustment for age and sex. Model 2 was further adjusted for marital status, educational level, smoking status, drinking status and WC. Model 3 additionally controlled for dyslipidemia, hypertension, cancer, TC, and CRP. P-value* for the pooled HR 95%CI. P-value^#^ for the heterogeneity.

Abbreviations: CMM, cardiometabolic multimorbidity; V1, visit 1.

Table S22 Multivariate Cox regression of the relationships between HbA1c/HDL-C ratio and the risk of incident CMM in ELSA and HRS after excluding participants with less than 2 years of follow-up

| **Characteristic** | **ELSA** | | | | | | **HRS** | | | | | |
| --- | --- | --- | --- | --- | --- | --- | --- | --- | --- | --- | --- | --- |
|  | **Model 1** | | **Model 2** | | **Model 3** | | **Model 1** | | **Model 2** | | **Model 3** | |
|  | **HR (95% CI)** | **P-value** | **HR (95% CI)** | **P-value** | **HR (95% CI)** | **P-value** | **HR (95% CI)** | **P-value** | **HR (95% CI)** | **P-value** | **HR (95% CI)** | **P-value** |
| **Cumulative HbA1c/HDL-C ratio** | | | | | | | | | | | | |
| **Per 1 SD** | 1.28 (1.13, 1.44) | <0.001 | 1.26 (1.11, 1.44) | <0.001 | 1.21 (1.06, 1.39) | 0.006 | 1.36 (1.19, 1.56) | <0.001 | 1.29 (1.11, 1.49) | <0.001 | 1.35 (1.16, 1.57) | <0.001 |
| **Quartile** |  |  |  |  |  |  |  |  |  |  |  |  |
| **Q1** | Ref | | Ref | | Ref | | Ref | | Ref | | Ref | |
| **Q2** | 0.77 (0.53, 1.11) | 0.164 | 0.78 (0.54, 1.14) | 0.202 | 0.76 (0.52, 1.11) | 0.159 | 0.92 (0.59, 1.44) | 0.725 | 0.84 (0.53, 1.31) | 0.436 | 0.83 (0.53, 1.31) | 0.419 |
| **Q3** | 1.31 (0.93, 1.84) | 0.117 | 1.30 (0.91, 1.85) | 0.144 | 1.23 (0.87, 1.76) | 0.246 | 1.31 (0.86, 2.00) | 0.209 | 1.11 (0.72, 1.71) | 0.641 | 1.10 (0.71, 1.70) | 0.674 |
| **Q4** | 1.53 (1.09, 2.14) | 0.014 | 1.48 (1.02, 2.13) | 0.037 | 1.33 (0.90, 1.95) | 0.150 | 1.73 (1.15, 2.59) | 0.009 | 1.39 (0.90, 2.15) | 0.137 | 1.48 (0.94, 2.32) | 0.087 |
| **P for trend** |  | 0.001 |  | 0.005 |  | 0.037 |  | 0.002 |  | 0.051 |  | 0.033 |
| **HbA1c/HDL-C ratio at V1** | | | | | | | | | | | | |
| **Per 1 SD** | 1.21 (1.08, 1.36) | 0.001 | 1.19 (1.05, 1.35) | 0.005 | 1.13 (0.98, 1.30) | 0.099 | 1.32 (1.15, 1.51) | <0.001 | 1.24 (1.07, 1.43) | 0.004 | 1.32 (1.13, 1.54) | <0.001 |
| **Quartile** |  |  |  |  |  |  |  |  |  |  |  |  |
| **Q1** | Ref | | Ref | | Ref | | Ref | | Ref | | Ref | |
| **Q2** | 1.15 (0.81, 1.64) | 0.429 | 1.14 (0.80, 1.63) | 0.472 | 1.12 (0.78, 1.60) | 0.529 | 1.51 (0.97, 2.35) | 0.070 | 1.34 (0.86, 2.10) | 0.199 | 1.33 (0.85, 2.09) | 0.213 |
| **Q3** | 1.24 (0.87, 1.78) | 0.233 | 1.21 (0.84, 1.75) | 0.306 | 1.15 (0.79, 1.67) | 0.461 | 1.71 (1.10, 2.65) | 0.017 | 1.45 (0.93, 2.28) | 0.104 | 1.43 (0.91, 2.26) | 0.122 |
| **Q4** | 1.72 (1.22, 2.43) | 0.002 | 1.64 (1.13, 2.37) | 0.009 | 1.43 (0.96, 2.15) | 0.081 | 1.89 (1.21, 2.93) | 0.005 | 1.52 (0.96, 2.42) | 0.076 | 1.66 (1.03, 2.70) | 0.039 |
| **P for trend** |  | 0.002 |  | 0.008 |  | 0.095 |  | 0.005 |  | 0.087 |  | 0.043 |
| **Trajectory of HbA1c/HDL-C ratio** | | | | | | | | | | | | |
| **Persistently low trajectory** | Ref | | Ref | | Ref | | Ref | | Ref | | Ref | |
| **Persistently high trajectory** | 1.68 (1.15, 2.47) | 0.008 | 1.57 (1.06, 2.33) | 0.025 | 1.52 (1.02, 2.25) | 0.040 | 2.42 (1.67, 3.52) | <0.001 | 2.18 (1.49, 3.18) | <0.001 | 2.51 (1.68, 3.75) | <0.001 |

Note: Model 1 included adjustment for age and sex. Model 2 was further adjusted for marital status, educational level, smoking status, drinking status and WC. Model 3 additionally controlled for dyslipidemia, hypertension, cancer, TC, and CRP.

Abbreviations: CMM, cardiometabolic multimorbidity; V1, visit 1.

Table S23 Pooled analysis of associations between HbA1c/HDL-C ratio and CMM risk in different models after excluding participants with less than 2 years of follow-up

| **Pooled analysis** | | | | | | | | | | | | |
| --- | --- | --- | --- | --- | --- | --- | --- | --- | --- | --- | --- | --- |
| **Characteristic** | **Model 1** | | | | **Model 2** | | | | **Model 3** | | | |
|  | **HR (95% CI)** | **P-value*** | **I^2^ (%)** | **P-value^#^** | **HR (95% CI)** | **P-value*** | **I^2^ (%)** | **P-value^#^** | **HR (95% CI)** | **P-value*** | **I^2^ (%)** | **P-value^#^** |
| **Cumulative HbA1c/HDL-C ratio** | | | | | | | | | | | | |
| **Per 1 SD** | 1.31 (1.20, 1.44) | <0.001 | 0.0 | 0.483 | 1.27 (1.16, 1.40) | <0.001 | 0.0 | 0.842 | 1.27 (1.15, 1.41) | <0.001 | 2.5 | 0.311 |
| **Quartile** |  |  |  |  |  |  |  |  |  |  |  |  |
| **Q1** | Ref | | | | Ref | | | | Ref | | | |
| **Q2** | 0.83 (0.62, 1.10) | 0.196 | 0.0 | 0.534 | 0.80 (0.60, 1.07) | 0.139 | 0.0 | 0.825 | 0.79 (0.59, 1.06) | 0.110 | 0.0 | 0.777 |
| **Q3** | 1.31 (1.01, 1.71) | 0.045 | 0.0 | 0.995 | 1.22 (0.93, 1.60) | 0.153 | 0.0 | 0.576 | 1.18 (0.89, 1.55) | 0.244 | 0.0 | 0.688 |
| **Q4** | 1.61 (1.24, 2.08) | <0.001 | 0.0 | 0.653 | 1.44 (1.09, 1.90) | 0.011 | 0.0 | 0.837 | 1.39 (1.04, 1.86) | 0.027 | 0.0 | 0.716 |
| **P for trend** |  | <0.001 |  |  |  | <0.001 |  |  |  | 0.003 |  |  |
| **HbA1c/HDL-C ratio at V1** | | | | | | | | | | | | |
| **Per 1 SD** | 1.26 (1.15, 1.37) | <0.001 | 0.0 | 0.356 | 1.21 (1.10, 1.33) | <0.001 | 0.0 | 0.719 | 1.22 (1.04, 1.42) | 0.012 | 52.2 | 0.148 |
| **Quartile** |  |  |  |  |  |  |  |  |  |  |  |  |
| **Q1** | Ref | | | | Ref | | | | Ref | | | |
| **Q2** | 1.28 (0.97, 1.69) | 0.080 | 0.0 | 0.353 | 1.21 (0.92, 1.61) | 0.173 | 0.0 | 0.575 | 1.20 (0.91, 1.59) | 0.205 | 0.0 | 0.560 |
| **Q3** | 1.42 (1.04, 1.93) | 0.026 | 18.2 | 0.269 | 1.30 (0.98, 1.73) | 0.069 | 0.0 | 0.540 | 1.26 (0.94, 1.68) | 0.121 | 0.0 | 0.468 |
| **Q4** | 1.78 (1.36, 2.34) | <0.001 | 0.0 | 0.744 | 1.59 (1.19, 2.12) | 0.002 | 0.0 | 0.809 | 1.52 (1.12, 2.08) | 0.008 | 0.0 | 0.643 |
| **P for trend** |  | <0.001 |  |  |  | 0.002 |  |  |  | 0.010 |  |  |
| **Trajectory of HbA1c/HDL-C ratio** | | | | | | | | | | | | |
| **Persistently low trajectory** | Ref | | | | Ref | | | | Ref | | | |
| **Persistently high trajectory** | 2.03 (1.42, 2.90) | <0.001 | 43.6 | 0.183 | 1.86 (1.35, 2.56) | <0.001 | 27.4 | 0.241 | 1.95 (1.19, 3.20) | 0.008 | 67.7 | 0.078 |

Note: Model 1 included adjustment for age and sex. Model 2 was further adjusted for marital status, educational level, smoking status, drinking status and WC. Model 3 additionally controlled for dyslipidemia, hypertension, cancer, TC, and CRP. P-value* for the pooled HR 95%CI. P-value^#^ for the heterogeneity.

Abbreviations: CMM, cardiometabolic multimorbidity; V1, visit 1.

Table S24 E-values for the association between HbA1c/HDL-C ratio and newly-onset CMM

|  | ELSA | HRS | Pooled |
| --- | --- | --- | --- |
| Cumulative HbA1c/HDL-C ratio | 1.61(1.34) | 2.13(1.76) | 1.99(1.67) |
| HbA1c/HDL-C ratio at V1 | 1.44(1.00) | 1.99(1.62) | 1.79(1.34) |
| Trajectory of HbA1c/HDL-C ratio | 2.22(1.45) | 4.07(2.75) | 3.41(2.26) |

Note: Data were expressed as E-value (E-value for lower limit of 95% CI). E-values were calculated based on hazard ratios from the fully adjusted model (Model 3).

Abbreviations: CMM, cardiometabolic multimorbidity; V1, visit 1.

Figure S1 Study design and timeline


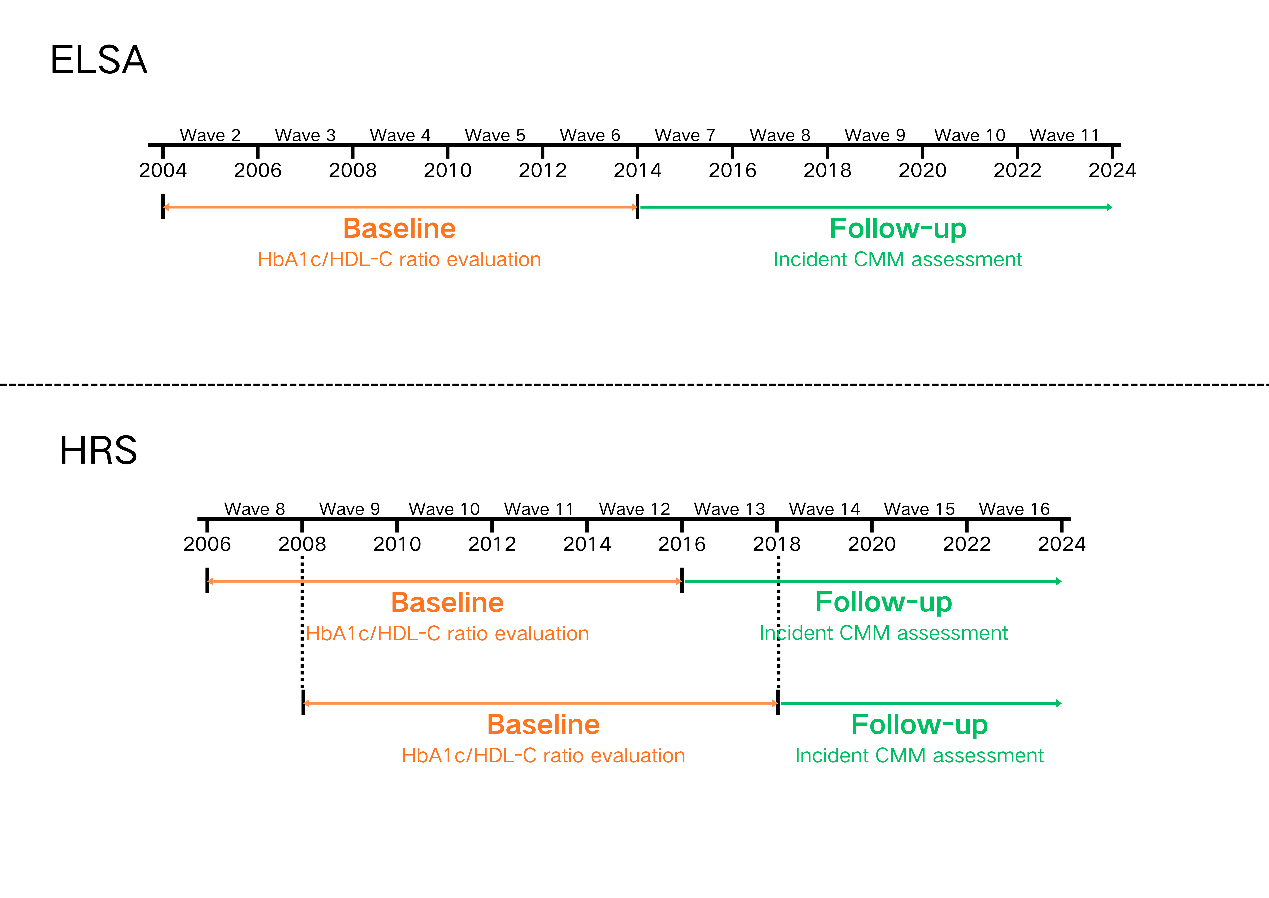


Abbreviations: CMM, cardiometabolic multimorbidity.

Figure S2 Subgroup and interaction analyses of the associations between HbA1c/HDL-C ratio at visit 1 and CMM risk.


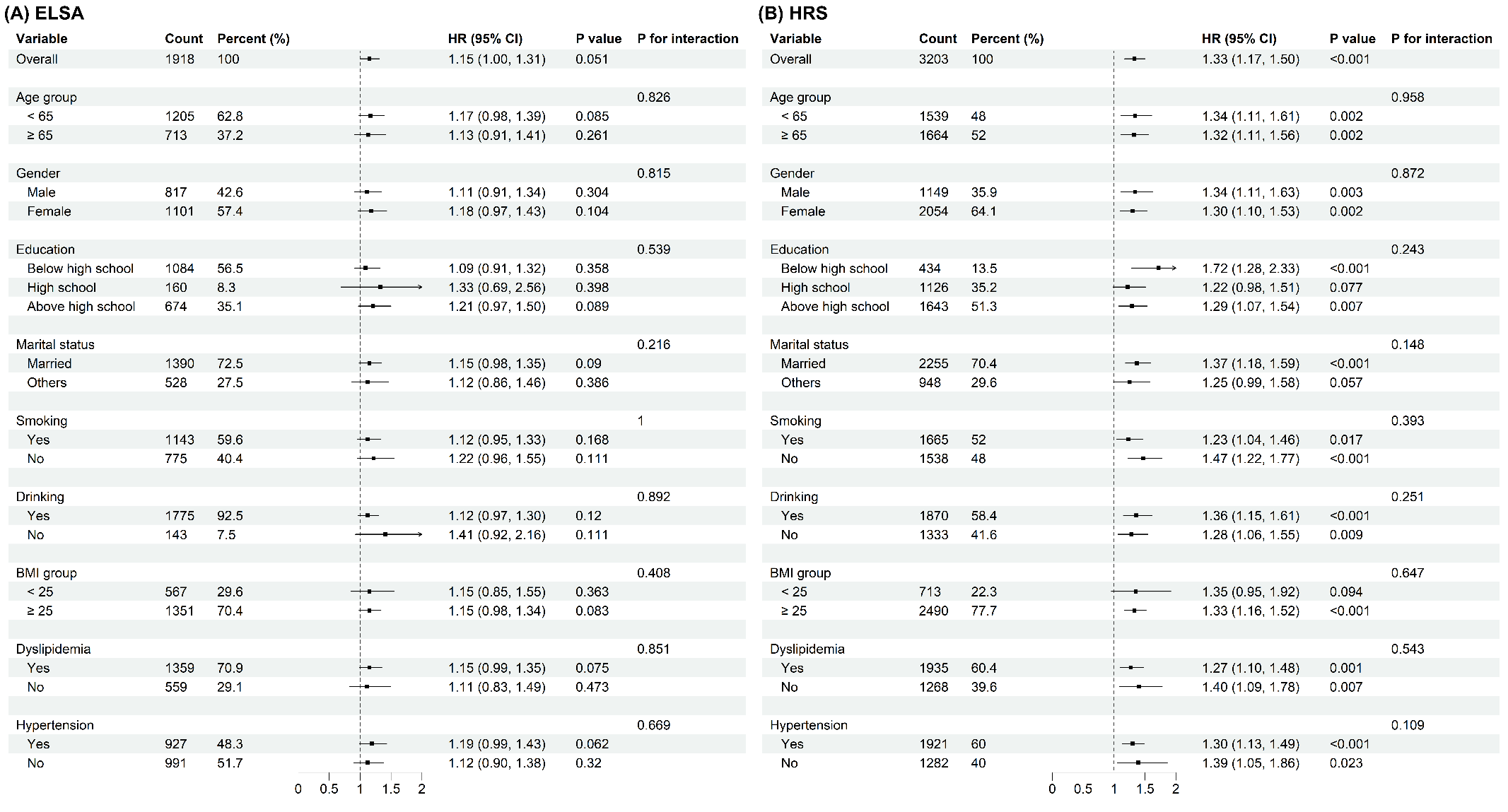


Note: The model was adjusted for age group, sex, marital status, educational level, smoking status, drinking status, WC, dyslipidemia, hypertension, cancer, TC, and CRP.

Abbreviation: CMM, cardiometabolic multimorbidity.

Figure S3 Subgroup and interaction analyses of the associations between trajectory of HbA1c/HDL-C ratio and CMM risk.


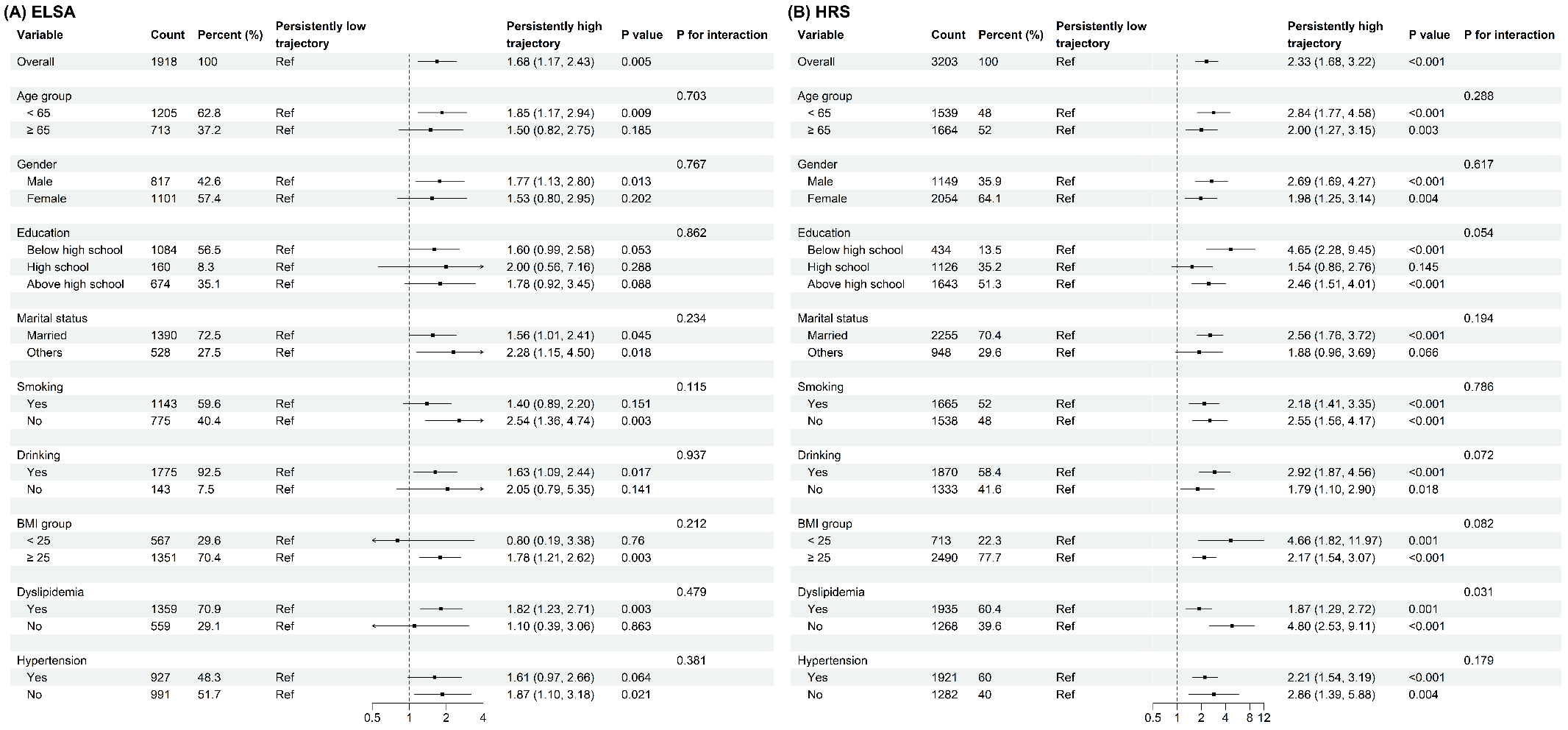


Note: The model was adjusted for age group, sex, marital status, educational level, smoking status, drinking status, WC, dyslipidemia, hypertension, cancer, TC, and CRP.

Abbreviation: CMM, cardiometabolic multimorbidity.
